# Pyrocatechol, a component of coffee, suppresses LPS-induced inflammatory responses by inhibiting NF-κB and activating Nrf2

**DOI:** 10.1038/s41598-020-59380-x

**Published:** 2020-02-13

**Authors:** Megumi Funakoshi-Tago, Yusuke Nonaka, Kenji Tago, Mika Takeda, Yuma Ishihara, Ami Sakai, Mari Matsutaka, Kenji Kobata, Hiroomi Tamura

**Affiliations:** 1 Department of Hygienic Chemistry, Faculty of Pharmacy, Keio University, 1-5-30 Shibakoen, Minato-ku, Tokyo 105-8512 Japan; 2 Division of Structural Biochemistry, Department of Biochemistry, Jichi Medical University, 3311-1 Yakushiji, Shimotsuke-shi, Tochigi-ken 329-0498 Japan; 3 Department of Pharmaceutical and Health Sciences, Josai University, 1-1 Keyakidai, Sakado, Saitama 350-0295 Japan

**Keywords:** Natural product synthesis, Nutrition, Molecular medicine

## Abstract

Coffee is a complex mixture of many bioactive compounds possessing anti-inflammatory properties. However, the mechanisms by which coffee exerts anti-inflammatory effects remains unclear and the active ingredients have not yet been identified. In this study, we found that coffee extract at more than 2.5%(v/v) significantly inhibited LPS-induced inflammatory responses in RAW264.7 cells and that anti-inflammatory activity of coffee required the roasting process. Interestingly, we identified pyrocatechol, a degradation product derived from chlorogenic acid during roasting, as the active ingredient exhibiting anti-inflammatory activity in coffee. HPLC analysis showed that 124 μM pyrocatechol was included in 100% (v/v) roasted coffee. A treatment with 5%(v/v) coffee extract and more than 2.5 μM pyrocatechol inhibited the LPS-induced activation of NF-κB and also significantly activated Nrf2, which acts as a negative regulator in LPS-induced inflammation. Furthermore, intake of 60% (v/v) coffee extract and 74.4 μM pyrocatechol, which is the concentration equal to contained in 60% (v/v) coffee, markedly inhibited the LPS-induced inflammatory responses in mice. Collectively, these results demonstrated that pyrocatechol, which was formed by the roasting of coffee green beans, is one of the ingredients contributing to the anti-inflammatory activity of coffee.

## Introduction

Inflammation is well understood as a critical biological response protecting the host’s body from pathogens to maintain tissue homeostasis. In the process of inflammation, macrophage play a central role in through its ability to produce pro-inflammatory mediators, such as nitric oxide (NO), cytokines, and chemokines^1,2^. A number of accumulated researches have evidenced that chronic inflammation is commonly involved in onsets of age-associated diseases, such as cardiovascular disease, diabetes, and cancer^3–5^. Therefore, the regulation of inflammatory responses is crucial for preventing malignancy of these inflammation-related diseases.

Lipopolysaccharide (LPS) constitutes outer membrane of Gram-negative bacteria, and well known to act as endotoxin triggering inflammatory responses through Toll-like receptor 4 (TLR4) on the surface of macrophages^6^. TLR4-mediated signals activate a transcription factor, nuclear factor kappaB (NF-κB), which mainly forms hetero-dimers including p65/RelA and p50 subunits. In the unstimulated cells, NF-κB binds to IκBα and forms inactive complex, in which IκBα masks nuclear localization signal (NES) included in p65/RelA subunit, leading the localization of inactive complex in cytosol. Stimulation with LPS causes the activation of IκB kinases (IKKs), and the phosphorylation of IκBα by IKKs triggers the degradation of IκBα mediated ubiquitin-proteasome system^7,8^. Then, released NF-κB is translocated into nucleus, and binds to the enhancer of its target genes by recognizing κB-responsive elements. NF-κB induces the expression of its target genes such as inducible nitric oxide synthase (iNOS) and inflammatory cytokines and chemokines, such as interleukin 6 (IL-6), tumor necrosis factor α (TNFα), CCL2, and CXCL1 through the transcriptional activity of NF-κB alone or its cooperation with other transcription factors such as activating protein-1 (AP-1) and nuclear factor of activated T cells (NFAT)^9–12^.

The transcription factor NF-E2-related factor-2 (Nrf2) is a critical transcription factor to protect cells from oxidative stresses by inducing the expression of many antioxidant genes. Several reports showed that disruption of the Nrf2 gene increased the mortality of mice in response to LPS-induced septic shock and caused severe allergen-driven air way inflammation in mice^13–15^. Furthermore, it was previously reported that Nrf2 interfered with the LPS-induced expression of IL-6 by ousting RNA polymerase II from IL-6 promoter^16^.

Coffee is widely consumed as beverage around the world, and its health promotion effects were reported by several studies. Especially, numerous epidemiological studies have reported a relationship between coffee consumption and reduced incidence of chronic diseases such as cancer, cardiovascular disease, obesity, and diabetes^17–21^. Coffee contains a number of compounds including caffeine, diterpenes, such as kahweol, and several polyphenols, such as chlorogenic acids^22,23^. Kahweol and chlorogenic acids were reported to suppress LPS-induced NO production and expression of IL-1β in murine macrophage cell line, RAW264.7 through the inhibition of NF-κB and JAK/STAT pathway, respectively^22,23^. The roasting procedure of coffee beans is required for the appearance of medical efficacy of coffee extract, and several parameters such as roasting temperature and periods affect the anti-inflammatory effects of coffee extracts in a mouse model in which septic shock was induced via a peritoneal injection of LPS^24^. These reports propose the possibility that consumption of coffee could prevent onset of chronic disease and effective ingredients restraining the onset of the chronic disease are included in coffee. However, it is still unclear how coffee extract suppresses the inflammatory responses including LPS signals, and furthermore, anti-inflammatory ingredients in coffee extract was not completely identified yet. Therefore, it is important to identify the active ingredients in coffee and analyze the mechanism action of identified ingredients.

In the present study, pyrocatechol formed by the roasting of coffee beans was identified as one of the coffee components that suppress LPS-induced inflammatory responses by inhibiting NF-κB and activating Nrf2.

## Results

### Coffee extract inhibited LPS-induced NO production and expression of CCL2, CXCL1, and IL-6

**Figure 1:**
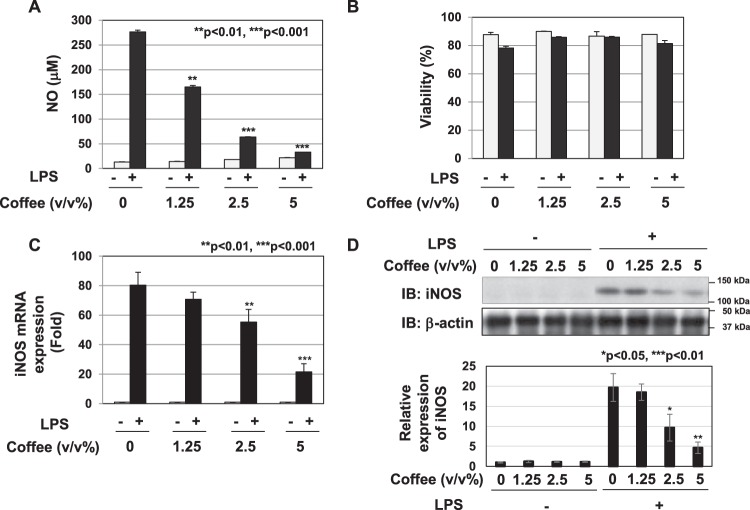
Extract of roasted coffee beans suppresses LPS-induced NO production and expression of iNOS. RAW264.7 cells were pretreated with various concentrations of coffee extract (1.25, 2.5, and 5% (v/v)) for 1 h prior to the stimulation with LPS (1 μg/mL). (**A**) The nitrate concentrations in culture supernatants were measured 24 h after the LPS stimulation using Griess reagent. **p < 0.01, ***p < 0.001 significantly different from control cells treated with LPS. (**B**) The cell viability was analyzed 24 h after the LPS stimulation using trypan blue exclusion tests. (**C**) iNOS mRNA expression was evaluated 12 h after the LPS stimulation by RT-PCR. GAPDH mRNA expression was used as an internal control. **p < 0.01, ***p < 0.001 significantly different from control cells treated with LPS. (**D**) Immunoblotting was performed 16 h after the LPS stimulation. The relative expression levels of iNOS are shown in the graph.

**Figure 2:**
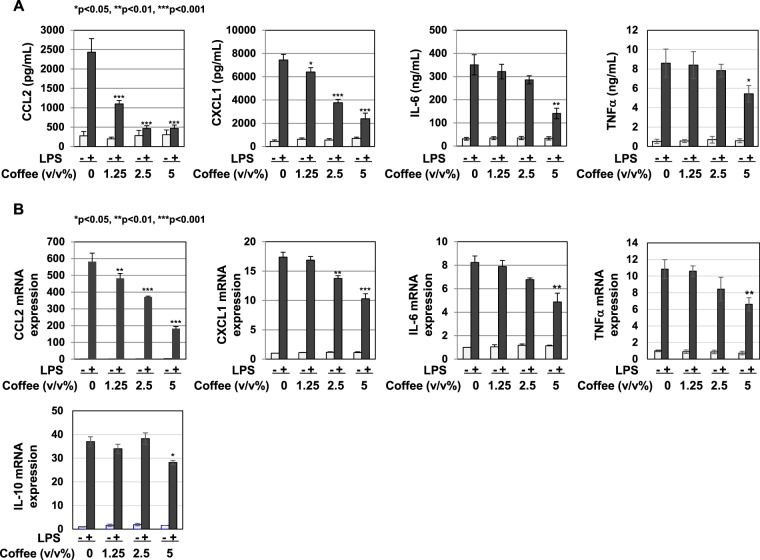
Extract of roasted coffee beans suppresses LPS-induced expression of CCL2, CXCL1, IL-6 and TNFα. RAW264.7 cells were pretreated with various concentrations of coffee extract (1.25, 2.5, and 5% (v/v)) for 1 h prior to the stimulation with LPS (1 μg/mL). (**A**) The amounts of CCL2, CXCL1, IL-6, and TNFα in supernatants were evaluated 24 h after the LPS stimulation by ELISA. *p < 0.05, **p < 0.01, ***p < 0.001 significantly different from control cells treated with LPS. (**B**) The mRNA expression of CCL2, CXCL1, IL-6, and TNFα was assessed 2 h after the LPS stimulation by RT-PCR. The expression of GAPDH mRNA was used as an internal control. **p < 0.01, ***p < 0.001 significantly different from control cells treated with LPS.

In order to analyze the anti-inflammatory activity of coffee extract, we investigated the effects of coffee extract on LPS-induced NO production in the murine macrophage cell line, RAW264.7. Coffee extract significantly inhibited the LPS-induced production of NO in a dose-dependent manner (Fig. 1A). Coffee extract exhibited little cytotoxicity at 5 (v/v)% or less when assessed using trypan blue exclusion tests (Fig. 1B). To clarify the mechanisms by which coffee extract inhibited NO production, we measured its effects on iNOS mRNA expression levels. The treatment with coffee extract reduced the LPS-induced expression of iNOS mRNA in a dose-dependent manner (Fig. 1C). Additionally, the LPS-induced expression of the iNOS protein was reduced by coffee extract (Fig. 1D). We also investigated whether coffee extract represses the expression of inflammation-related chemokines and cytokines induced by LPS. Coffee extract markedly inhibited the LPS-induced secretion of CCL2, CXCL1, IL-6, and TNFα (Fig. 2A). In addition, coffee extract significantly inhibited the LPS-induced mRNA expression of CCL2, CXCL1, IL-6, and TNFα (Fig. 2B). Coffee extract also inhibited the LPS-induced expression of IL-10, which is a representative anti-inflammatory cytokine (Fig. 2B).

### Coffee extract suppressed LPS-induced NF-κB activation by inhibiting IKK activation

**Figure 3:**
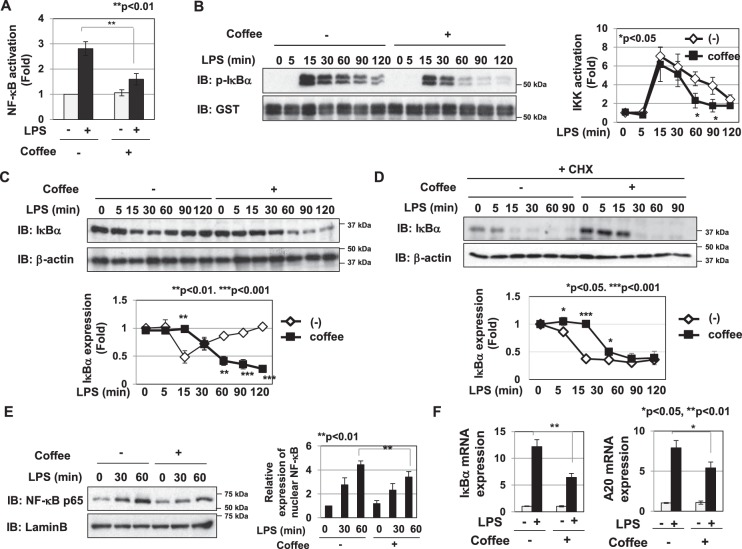
Extract of roasted coffee beans suppresses LPS-induced NF-κB activation by inhibiting IKK activation. (**A**) RAW264.7 cells were transfected with pNF-κB-Luc and pRL-TK as described in the Materials and methods. Transfected cells were pretreated with coffee extract (5% (v/v)) for 1 h prior to the LPS stimulation (1 μg/mL) for 6 h. NF-κB-dependent luciferase activity was normalized to the activity of constitutively expressed Renilla luciferase. (**B**–**F**) RAW264.7 cells were pretreated with coffee extract (5% (v/v)) for 1 h prior to the LPS stimulation (1 μg/mL) for the indicated periods. (**B**) IKK activity was analyzed using purified GST-IκBα as a substrate by *in vitro* kinase assay. Immunoblotting was performed using an anti-phospho-IκBα or anti-GST antibody. The relative IKK activity was shown in the graph. (**C**,**D**) In the absence and presence of cycloheximide (CHX), whole cells lysates were prepared and immunoblotting was performed by using an anti-IκBα or anti-β-actin antibody. The relative protein amounts of IκBα was show in in graphs. (**E**) Nuclear extracts were prepared and immunoblotted with an anti-NF-κB p65 or anti-Lamin B antibody. The relative expression level of NF-κB in the nucleus was shown in the graph. (**F**) The mRNA expression of IκBα and A20 was assessed 2 h after the LPS stimulation by RT-PCR. The expression of GAPDH mRNA was used as an internal control. *p < 0.05, **p < 0.01 significantly different from control cells treated with LPS.

The transcription factor NF-κB regulates the expression of iNOS, CCL2, CXCL1, IL-6, and TNFα^9–12^. The present results showed that DHMEQ, a NF-κB inhibitor, markedly suppressed LPS-induced NO production and the mRNA expression of iNOS, CCL2, CXCL2, IL-6, and TNFα in RAW264.7cells (Supplemental Fig. 1). Thus, we examined the effects of coffee extract on the LPS-induced transcriptional activation of NF-κB using a luciferase assay. Coffee extract significantly inhibited LPS-induced NF-κB activation (Fig. 3A). In order to gain further insights into the mechanisms by which coffee extract inhibits LPS-induced NF-κB activation, we examined its effects on LPS-induced IKK activation. Coffee extract significantly suppressed LPS-induced IKK activation (Fig. 3B). The activation of IKK was observed 15 min after the stimulation with LPS, and then slowly decayed (Left half in Fig. 3B). When treated with coffee extract, the activation of IKK was similarly maximized 15 min after the LPS stimulation as a control experiment; however, IKK activity diminished more quickly than under the condition without the coffee treatment (Right half in Fig. 3B). We then examined the effects of coffee extract on LPS-induced IκBα degradation in the absence and presence of cycloheximide (CHX). Whereas IκBα was degraded 15 min after the stimulation with LPS, and the protein expression of IκBα then recovered (Left half in Fig. 2C), the treatment with coffee extract delayed the degradation of IκBα and suppressed the new synthesis of the IκBα protein (Right half in Fig. 2C). In the presence of CHX, whereas the expression of IκBα was completely decreased 15 min after the stimulation with LPS (Left half in Fig. 3D), the treatment with coffee extract delayed the degradation of IκBα until 30 min after stimulation with LPS (Right half in Fig. 3D). We then investigated the nuclear localization of the p65/RelA NF-κB subunit. As shown in Fig. 3E, the treatment with coffee effectively suppressed the nuclear localization of p65/RelA. A20 and IκBα are the target genes of NF-κB^25–27^. Coffee extract significantly inhibited the LPS-induced mRNA expression of IκBα and A20 (Fig. 3F).

### Coffee extract had no effect on the LPS-induced activation of JNK and p38

**Figure 4:**
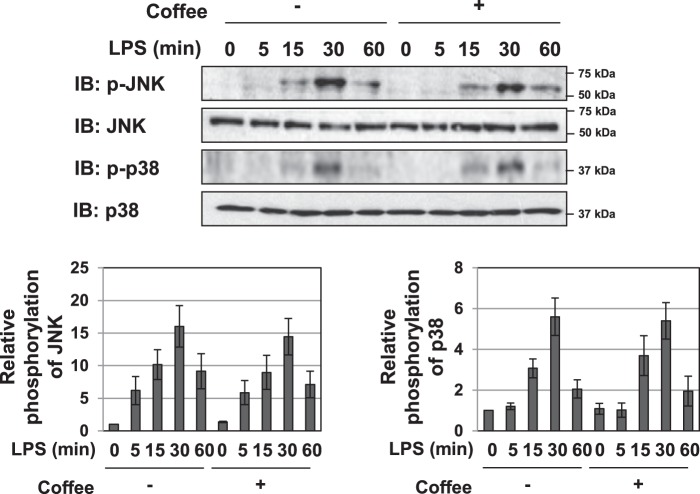
Extract of roasted coffee beans had no effect on the LPS-induced activation of JNK and p38. RAW264.7 cells were pretreated with coffee extract (5% (v/v)) for 1 h prior to the LPS stimulation (1 μg/mL) for the indicated periods. Whole cell lysates were immunoblotted with antibodies against phospho-JNK, JNK, phospho-p38, and p38. The relative phosphorylation levels of JNK and p38 were shown in the graphs.

Previous studies reported that LPS induces the activation of the MAP kinase pathway, such as JNK and p38, as well as the NF-κB pathway to facilitate inflammation^28,29^. The present results demonstrated that specific inhibitors against JNK and p38, SB203580 and SP600125, also suppressed the LPS-induced expression of iNOS and inflammation-related cytokines (Supplemental Fig. 2), suggesting the involvement of JNK and p38 in the expression of these genes. We examined the effects of coffee extract on the LPS-induced phosphorylation of JNK and p38 using each phospho-specific antibody; however, LPS-induced activation remained unchanged in RAW264.7 cells (Fig. 4).

### Coffee extract induced the expression of Nrf2, which negatively regulated LPS-induced inflammatory responses

**Figure 5:**
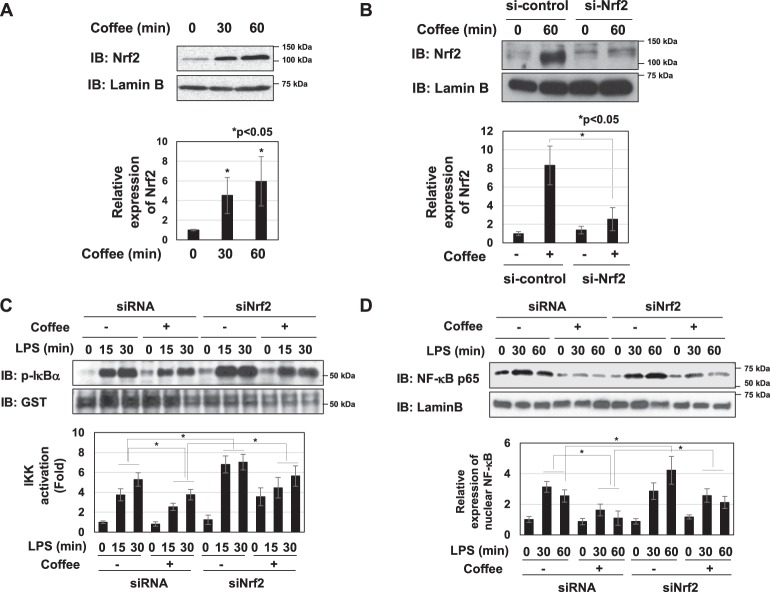
Extract of roasted coffee beans induces the expression of Nrf2, which negatively inhibits LPS-induced activation of IKK and nuclear translocation of NF-κB. (**A**,**B**) RAW264.7 cells and transfected RAW264.7 cells with control siRNA (si-control) or siRNA against Nrf2 (si-Nrf2) were treated with coffee extract (5% (v/v)) for the indicated periods. Nuclear extracts were immunoblotted with an anti-Nrf2 or anti-Lamin B antibody. The relative expression levels of Nrf2 in the nucleus are shown in the graphs. (**C**,**D**) Transfected RAW264.7 cells were treated with coffee extract (5% (v/v)) for 1 h prior to LPS stimulation for the indicated periods. (**C**) IKK activity was analyzed by *in vitro* kinase assay and the relative IKK activity was shown in the graph. (**D**) Nuclear extracts were immunoblotted with an anti-NF-κB p65 or anti-Lamin B antibody. The relative expression level of NF-κB in the nucleus was shown in the graph.

**Figure 6:**
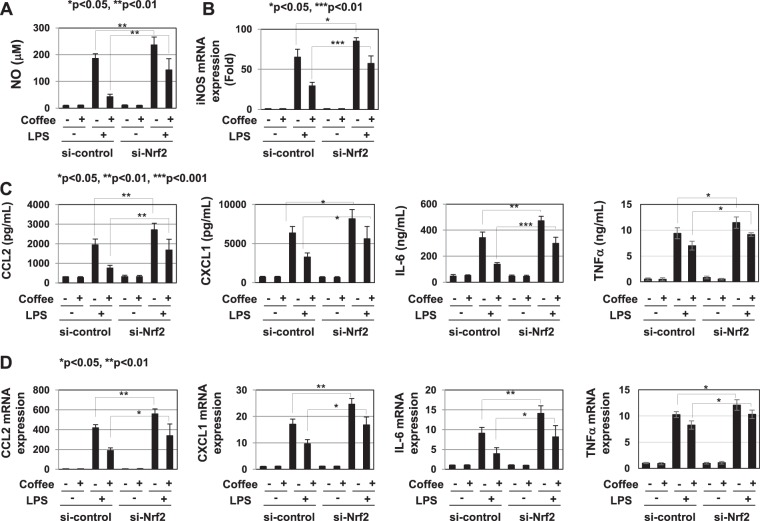
Extract of roasted coffee beans induces the expression of Nrf2, which negatively inhibits LPS-induced inflammatory responses. Transfected RAW264.7 cells with control siRNA (si-control) or siRNA against Nrf2 (si-Nrf2) were pretreated with coffee extract (5% (v/v)) for 1 h prior to the stimulation with LPS (1 μg/mL) for the indicated periods. (**A**) Nitrate concentrations in culture supernatants were measured 24 h after the LPS stimulation using Griess reagent. (**B**) iNOS mRNA expression was evaluated 12 h after the LPS stimulation by RT-PCR. GAPDH mRNA expression was used as an internal control. (**C**) The amounts of CCL2, CXCL1, IL-6, and TNFα in supernatants were evaluated 24 h after the LPS stimulation by ELISA. (**D**) The mRNA expression of CCL2, CXCL1, IL-6, and TNFα was assessed 2 h after the LPS stimulation by RT-PCR. The expression of GAPDH mRNA was used as an internal control.

We attempted to elucidate the mechanisms by which coffee extract suppresses LPS-induced NF- κB activation. Previous studies reported that the transcription factor, Nrf2 negatively regulated inflammation, and suggested the involvement of the suppression of NF-κB^14–16^. Coffee extract markedly induced the expression of Nrf2 in RAW264.7 cells (Fig. 5A). When Nrf2 was knocked down in RAW264.7 cells, LPS-induced IKK activation and nuclear localization of p65/RelA NF-κB subunit was enhanced and the inhibitory effects of coffee extract on them were reduced (Fig. 5B–D). The knockdown of Nrf2 increased the LPS-induced NO production and iNOS mRNA expression and reduced the inhibitory effects of coffee extract on them (Fig. 6A,B). Furthermore, the knockdown of Nrf2 increased the LPS-induced secretion of CCL2, CXCL1, IL-6, and TNFα and the mRNA expression of CCL2, CXCL1, IL-6, and TNFα, and reduced the inhibitory effects of coffee extract on their secretion and expression (Fig. 6C,D).

### The anti-inflammatory activity of coffee extract was reinforced depending on the roasting period of coffee beans

**Figure 7:**
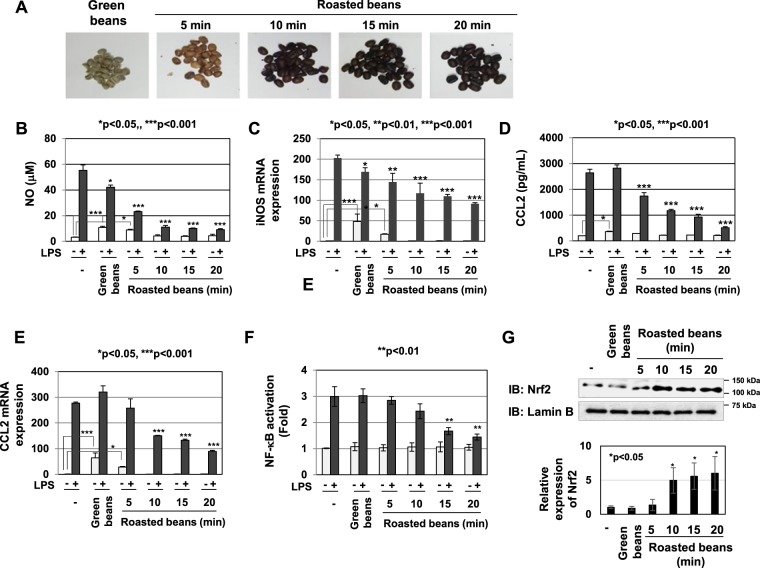
Anti-inflammatory activity of coffee bean extract depends on the roasting degree. (**A**) Green coffee beans were roasted at 220 °C for the indicated periods. (**B**–**E**) RAW264.7 cells were pretreated with extracts of coffee beans roasted for various periods (5% (v/v)) for 1 h prior to the stimulation with LPS (1 μg/mL). (**B**) Nitrate concentrations in culture supernatants were measured 24 h after the LPS stimulation using Griess reagent. (**C**) iNOS mRNA expression was evaluated 12 h after the LPS stimulation by RT-PCR. GAPDH mRNA expression was used as an internal control. (**D**) The amounts of CCL2 in supernatants were evaluated 24 h after the LPS stimulation by ELISA. (**E**) CCL2 mRNA expression was assessed 2 h after LPS stimulation by RT-PCR. The expression of GAPDH mRNA was used as an internal control. (**F**) RAW264.7 cells were transfected with pNF-κB-Luc and pRL-TK. Transfected cells were pretreated with each coffee extract (5% (v/v)) for 1 h prior to the LPS stimulation (1 μg/mL) for 6 h. NF-κB-dependent luciferase activity was normalized to the activity of constitutively expressed Renilla luciferase. (**G**) RAW264.7 cells were treated with each coffee extract (5% (v/v)) for 1 h. Nuclear extracts were immunoblotted with an anti-Nrf2 or anti-Lamin B antibody. The relative expression levels of Nrf2 in the nucleus are shown in the graph.

Previous studies reported the requirement of a number of procedures, such as the roasting of coffee beans, to obtain their medicinal effects^24^. To clarify whether the roasting procedure is required for the anti-inflammatory effects of coffee extract, we roasted green coffee beans at 220 degrees for different periods (5, 10, 15, and 20 min) and prepared their extracts (Fig. 7A). The extract of green coffee beans significantly induced NO production and iNOS mRNA expression regardless of the LPS stimulation, and slightly inhibited LPS-induced NO production and iNOS mRNA expression (Fig. 7B,C). The inhibitory effects of coffee extract on NO production and iNOS mRNA expression induced by LPS was reinforced in a manner that depended on the length of the roasting period (Fig. 7B,C). The extract of green coffee beans also induced CCL2 secretion and CCL2 mRNA expression regardless of the LPS stimulation; however, the inhibitory effects of coffee extract on LPS-induced CCL2 secretion and CCL2 mRNA expression were reinforced in a manner that depended on the degree of roasting (Fig. 7D,E). Furthermore, the extract of green coffee beans failed to inhibit LPS-induced NF-κB activation or induce Nrf2 expression, while the extract of roasted coffee beans significantly inhibited LPS-induced NF-κB activation and induced Nrf2 expression (Fig. 7F,G).

### The ethyl acetate fraction (E) of coffee extract exhibited anti-inflammatory activity in RAW264.7 cells

**Figure 8:**
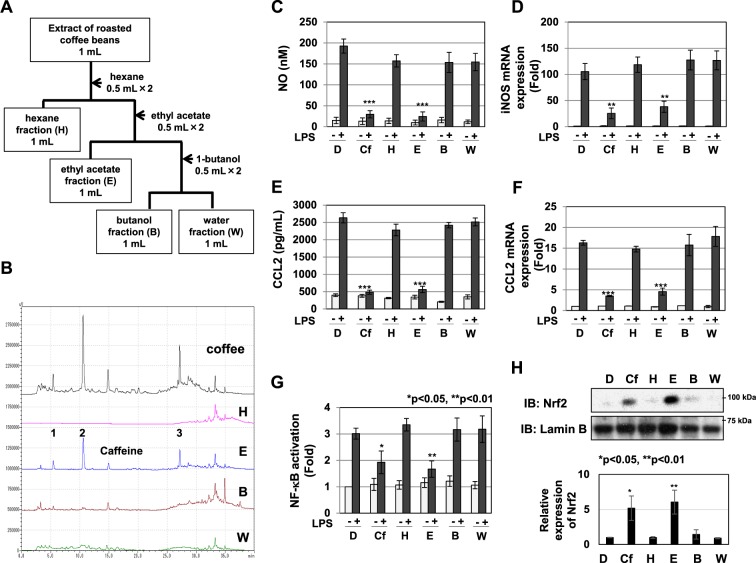
The ethyl acetate fraction prepared from the extract of roasted coffee beans exhibits anti-inflammatory activity. (**A**) Procedure for the fractionation of coffee extract using organic solvents. (**B**) The extract of roasted coffee beans and each fraction were analyzed by HPLC. (**C**-**F**) RAW264.7 cells were pretreated with coffee extract (5% (v/v) (Cf) and each fraction equivalent to 5% (v/v) of coffee (H: hexane fraction, E: ethyl acetate fraction, B: butanol fraction, W: water fraction) for 1 h prior to the LPS stimulation (1 μg/mL). (**C**) Nitrate concentrations in culture supernatants were measured 24 h after the LPS stimulation using Griess reagent. (**D**) iNOS mRNA expression was evaluated 12 h after the LPS stimulation by RT-PCR. GAPDH mRNA expression was used as an internal control. (**E**) The amounts of CCL2 in supernatants were evaluated 24 h after the LPS stimulation by ELISA. (**F**) CCL2 mRNA expression was assessed 2 h after the LPS stimulation by RT-PCR. The expression of GAPDH mRNA was used as an internal control. (**G**) RAW264.7 cells were transfected with pNF-κB-Luc and pRL-TK. Transfected cells were pretreated with coffee extract (5% (v/v)) and each fraction equivalent to 5% (v/v) of coffee for 1 h prior to LPS stimulation for 6 h. NF-κB-dependent luciferase activity was normalized to the activity of constitutively expressed Renilla luciferase. (**H**) RAW264.7 cells were treated with coffee extract (5% (v/v)) and each fraction equivalent to 5% (v/v) of coffee for 1 h. Nuclear extracts were immunoblotted with an anti-Nrf2 or anti-Lamin B antibody. The relative expression levels of Nrf2 in the nucleus are shown in the graph.

**Figure 9:**
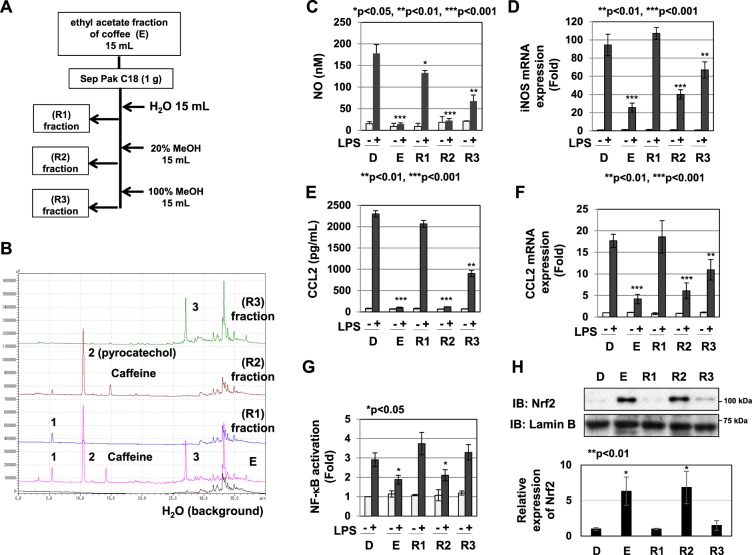
The R2 fraction prepared from the ethyl acetate fraction of the roasted coffee bean extract exhibits anti-inflammatory activity. (**A**) Procedure for the fractionation of the ethyl acetate fraction of coffee bean extract using the Sep Pak C18 column. (**B**) The ethyl acetate fraction of the roasted coffee bean extract (**E**) and the R1-R3 fractions were analyzed by HPLC. (**C**–**F**) RAW264.7 cells were pretreated with fractions R1-R3, equivalent to 5% (v/v) of coffee, for 1 h prior to the LPS stimulation (1 μg/mL). (**C**) Nitrate concentrations in culture supernatants were measured 24 h after the LPS stimulation using Griess reagent. (**D**) iNOS mRNA expression was assessed 12 h after the LPS stimulation by RT-PCR. GAPDH mRNA expression was used as an internal control. (**E**) The amounts of CCL2 in supernatants were evaluated 24 h after the LPS stimulation by ELISA. (**F**) CCL2 mRNA expression was assessed 2 h after the LPS stimulation by RT-PCR. (**G**) RAW264.7 cells were transfected with pNF-κB-Luc and pRL-TK. Transfected cells were pretreated with fractions R1-R3, equivalent to 5% (v/v) of coffee, for 1 h prior to the stimulation with LPS (1 μg/mL) for 6 h. NF-κB-dependent luciferase activity was normalized to the activity of constitutively expressed Renilla luciferase. (**H**) RAW264.7 cells were treated with fractions R1-R3, equivalent to 5% (v/v) of coffee, for 1 h. Nuclear extracts were immunoblotted with an anti-Nrf2 or anti-Lamin B antibody. The relative expression levels of Nrf2 in the nucleus are shown in the graph.

As shown above, the anti-inflammatory activity of coffee extract needs procedures such as roasting, suggesting the requirement of component alterations caused by chemical reactions. To identify the ingredients that exhibit anti-inflammatory activity in roasted coffee beans, we progressively fractionated the roasted coffee extract using hexane, ethyl acetate, and butanol, as shown in Fig. 8A. Each condition of solvent-extraction showed different HPLC elution patterns of the components (Fig. 8B). When We then examined the anti-inflammatory activity of each organic solvent fraction, the ethyl acetate fraction (E) of coffee extract markedly inhibited LPS-induced NO production and iNOS mRNA expression (Fig. 8C,D). It also markedly inhibited LPS-induced CCL2 secretion and CCL2 mRNA expression (Fig. 8E,F), and significantly inhibited LPS-induced NF-κB activation and induced Nrf2 expression (Fig. 8G,H). The E fraction was further fractioned using the ODS column into three fractions: R1, R2, and R3, as shown in Fig. 9A,B. We then examined the anti-inflammatory activities of the R1, R2, and R3 fractions. The R2 fraction markedly inhibited LPS-induced NO production and iNOS mRNA expression, while the R3 fraction slightly inhibited LPS-induced NO production and iNOS mRNA expression (Fig. 9C,D). The R2 fraction markedly inhibited, while the R3 fraction slightly inhibited LPS-induced CCL2 secretion and CCL2 mRNA expression (Fig. 9E,F). On the other hand, the R1 fraction had no effect on LPS-induced NO production, iNOS mRNA expression, CCL2 secretion, or CCL2 mRNA expression (Fig. 9C–F). Furthermore, the R2 fraction significantly inhibited LPS-induced NF-κB activation and induced Nrf2 expression (Fig. 9G,H).

### Pyrocatechol, a component of roasted coffee, exhibited anti-inflammatory activity in RAW264.7 cells

**Figure 10:**
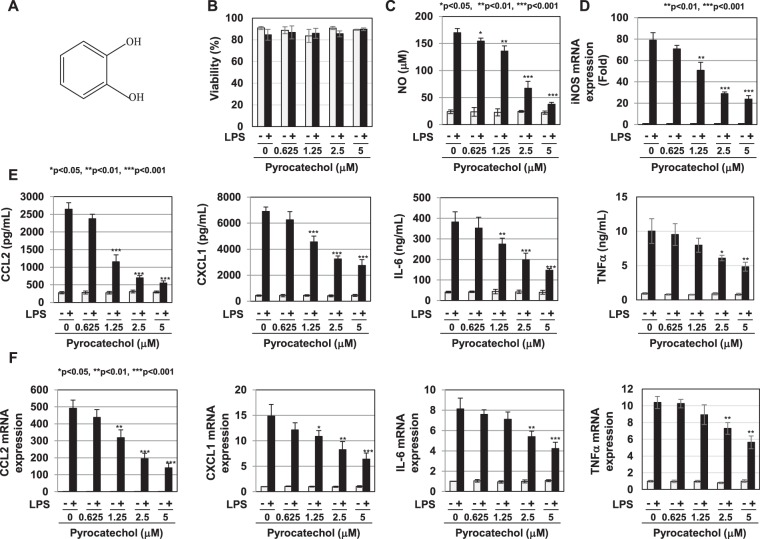
Pyrocatechol in the R2 fraction exhibits anti-inflammatory activity. (**A**) Structure of pyrocatechol. (**B**–**F**) RAW264.7 cells were pretreated with pyrocatechol (0.625, 1.25, 2.5, and 5 μM) for 1 h prior to the LPS stimulation (1 μg/mL). (**B**) Viability was assessed 24 h after the LPS stimulation using trypan blue exclusion tests. (**C**) Nitrate concentrations in culture supernatants were measured 24 h after the LPS stimulation using Griess reagent. (**D**) The expression of iNOS mRNA was assessed 12 h after the LPS stimulation by RT-PCR. GAPDH mRNA expression was used as an internal control. (**E**) The amounts of CCL2, CXCL1, IL-6, and TNFα in supernatants were evaluated 24 h after the LPS stimulation by ELISA. (**F**) The mRNA expression of CCL2, CXCL1, IL-6, and TNFα was assessed 2 h after the LPS stimulation by RT-PCR. GAPDH mRNA expression was used as an internal control.

**Figure 11:**
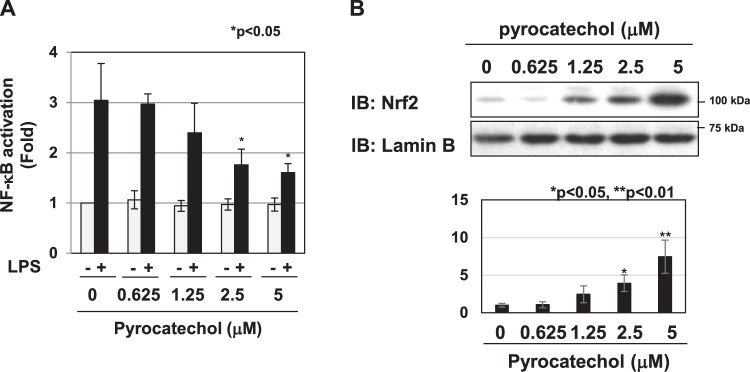
Pyrocatechol inhibits LPS-induced NF-κB activation and activates Nrf2. (**A**) RAW264.7 cells were transfected with pNF-κB-Luc and pRL-TK. Transfected cells were pretreated with pyrocatechol (0.625, 1.25, 2.5, and 5 μM) for 1 h prior to the stimulation with LPS (1 μg/mL) for 6 hr. NF-κB-dependent luciferase activity was normalized to the activity of constitutively expressed Renilla luciferase. (**B**) RAW264.7 cells were pretreated with pyrocatechol (0.625, 1.25, 2.5, and 5 μM) for 1 h. Nuclear extracts were immunoblotted with an anti-Nrf2 or anti-Lamin B antibody. The relative expression levels of Nrf2 in the nucleus are shown in the graph.

The HPLC analysis revealed pyrocatechol as the active ingredient in the R2 fraction of the E fraction of coffee extract (Fig. 10A). While 2.39 mM of caffeine, a well-known component in coffee, was included in 100% (v/v) of roasted coffee, a smaller amount (0.124 mM) of pyrocatechol was present. As shown in Fig. 10B, pyrocatechol exhibited little cytotoxicity at 5 μM or less. In order to clarify whether pyrocatechol exhibits anti-inflammatory activity, we examined the effects of pyrocatechol on LPS-induced inflammatory responses. Pyrocatechol significantly inhibited LPS-induced NO production and iNOS mRNA expression in a dose-dependent manner (Fig. 10C,D). In addition, the treatment with pyrocatechol markedly reduced the LPS-induced secretion of CCL2, CXCL1, IL-6, and TNFα and mRNA expression of CCL2, CXCL1, IL-6, and TNFα (Fig. 10E,F). On the other hand, the treatment with pyrocatechol had no effect on LPS-induced expression of IL-10 mRNA (Supplemental Fig. 5A). We then examined whether pyrocatechol contained in coffee exhibited anti-inflammatory activity using the same mechanism of action as coffee. Pyrocatechol significantly inhibited LPS-induced NF-κB activation (Fig. 11A) and induced the expression of Nrf2 (Fig. 11B). Pyrocatechol and coffee extract did not alter the mRNA expression of Nrf2 (Supplemental Fig. 3A,C). The transcriptional activation of Nrf2 was induced by the treatment with coffee extract and pyrocatechol, evidenced by increase in expression of its target genes HO-1 and NQO1 and HO-1 protein (Supplemental Fig. 3B,D,E).

### Coffee extract and pyrocatechol inhibited LPS-induced inflammation in mice

**Figure 12:**
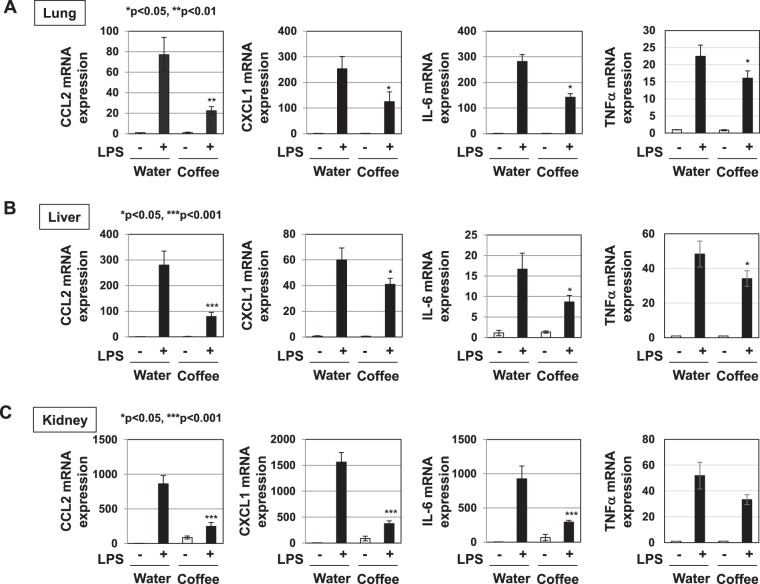
Administration of the extract of roasted coffee beans significantly prevents the LPS-induced expression of CCL2, CXCL1, IL-6, and TNFα in mice. Male C57BL/6 mice were given water or 60% (v/v) coffee extract for 3 weeks and then injected intraperitoneally with PBS or LPS (200 μg/mouse) (6 mice per group). Seven hours after the injection of PBS or LPS, total RNA was extracted from the lungs (**A**), liver (**B**), and kidneys (**C**). The mRNA expression of CCL2, CXCL1, IL-6, and TNFα was assessed by RT-PCR. GAPDH mRNA was analyzed as an internal control. Values are means ± SD from six independent experiments.

We investigated whether coffee extract and pyrocatechol inhibit the LPS signaling pathway *in vivo*. In previous study, we reported that until 60% (v/v) coffee did not affect the drinking intake and food intake compared to water, when C57BL/6 mice were allowed free access to coffee extract with different coffee extracts^30^. Thus, C57BL/6 mice were given regular drinking water (control) or 60% (v/v) coffee extract for 3 weeks. No significant differences were observed between the intake amounts of coffee extract and water (Supplemental Fig. 4A). The intake of coffee extract did not affect the amounts of food consumed or increases in body weights (Supplemental Fig. 4B,C). We observed that the mRNA expression of CCL2, CXCL1, IL-6, and TNFα in lungs were induced 7 h after LPS injection (Supplemental Fig. 6). Therefore, after 3 weeks, mice were intraperitoneally injected with control vehicle, PBS or LPS and total RNA was extracted from the lungs, liver, and kidneys of each mouse 7 h after injection. Whereas the mRNA expression of CCL2, CXCL1, IL-6, and TNFα in lungs, liver and kidneys was increased by LPS stimulation, the preadministration of coffee extract significantly decreased the LPS-induced mRNA expression of these cytokines in each organ (Fig. 12A–C).

**Figure 13:**
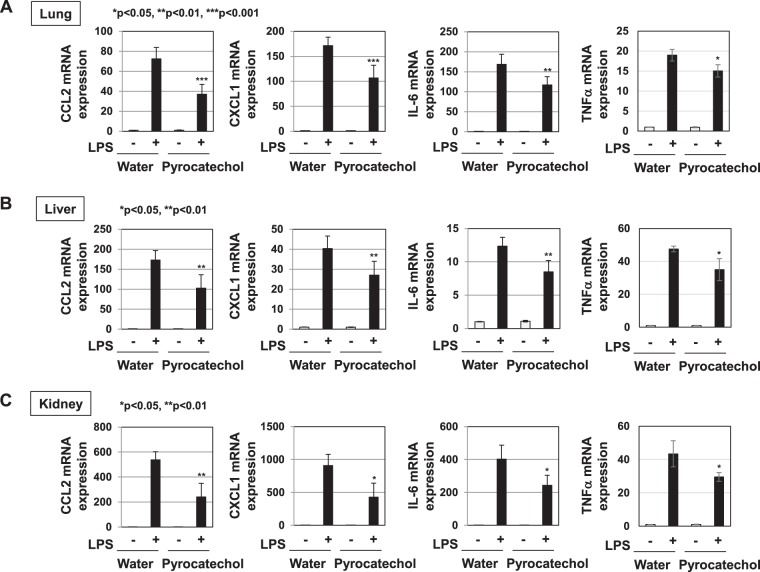
Administration of pyrocatechol significantly prevents the LPS-induced expression of CCL2, CXCL1, IL-6, and TNFα in mice. Male C57BL/6 mice were given water or 74.4 μM of pyrocatechol, which is equivalent concentration to the content of pyrocatechol in 60% (v/v) coffee, for 3 weeks and then injected intraperitoneally with PBS or LPS (200 μg/mouse) (6 mice per group). Seven hours after the injection of PBS or LPS, total RNA was extracted from the lungs (**A**), liver (**B**), and kidneys (**C**). The mRNA expression of CCL2, CXCL1, and IL-6 was assessed by RT-PCR. GAPDH mRNA was analyzed as an internal control. Values are means ± SD from six independent experiments. *, **, and ***indicate *p* < 0.05, *p* < 0.01, and *p* < 0.001, respectively.

**Figure 14:**
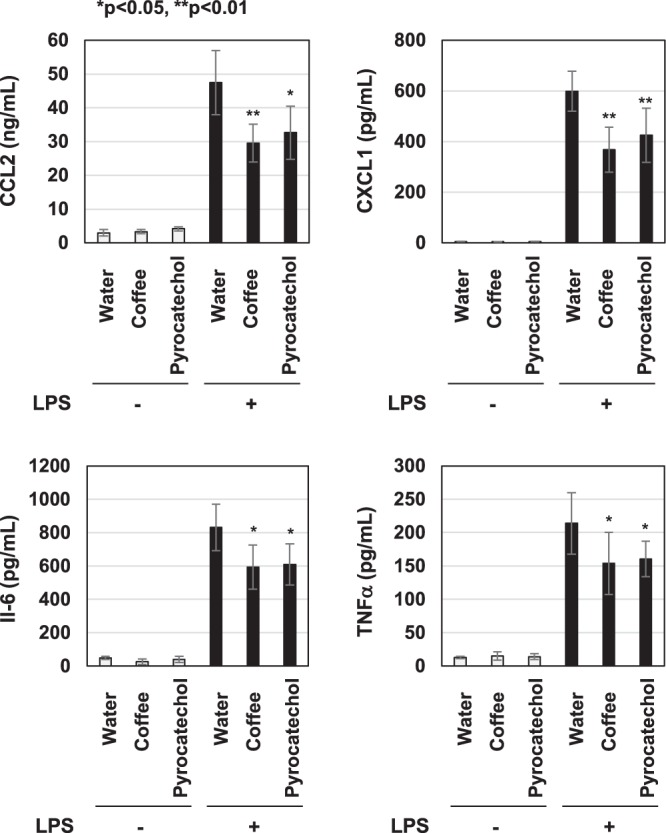
Administration of the extract of roasted coffee beans and pyrocatechol significantly reduced LPS-induced serum level of CCL2, CXCL1, IL-6, and TNFα in mice. Male C57BL/6 mice were given water, 60% (v/v) coffee extract or 74.4 μM of pyrocatechol for 3 weeks and then injected intraperitoneally with PBS or LPS (200 μg/mouse) (6 mice per group). One hours after the LPS injection, blood samples from mice were collected by cardiac puncture. The amounts of CCL2, CXCL1, IL-6, and TNFα in murine serum were measured by ELISA. *, **, and ***indicate *P* < 0.05, *P* < 0.01, and *P* < 0.001, respectively.

The concentration of pyrocatechol which is contained in 60% (v/v) coffee is 74.4 μM. We then performed a similar experiment *in vivo* in which we administered 74.4 μM pyrocatechol. The consumption of pyrocatechol per day was 49.2 ± 2.8 μg. The addition of pyrocatechol into drinking water did not affect the intake amount of water, food consumption, or increases in body weight (Supplemental Fig. 4D–F). The administration of pyrocatechol also significantly reduced the LPS-induced mRNA expression of CCL2, CXCL1, IL-6, and TNFα in the lungs, liver, and kidneys (Fig. 13A–C). On the other hand, the LPS-induced expression of IL-10 mRNA in these organs was not affected by administration of coffee extract and pyrocatechol (Supplemental Fig. 5B,C). Furthermore, we investigated whether administration with coffee and pyrocatechol could inhibit the LPS-induced production of CCL2, CXCL-1, IL-6 and TNFα in serum level. In mice administrated with coffee and pyrocatechol, serum levels of CCL2, CXCL-1, IL-6 and TNFα induced by LPS injection was significantly decreased (Fig. 14).

## Discussion

**Figure 15:**
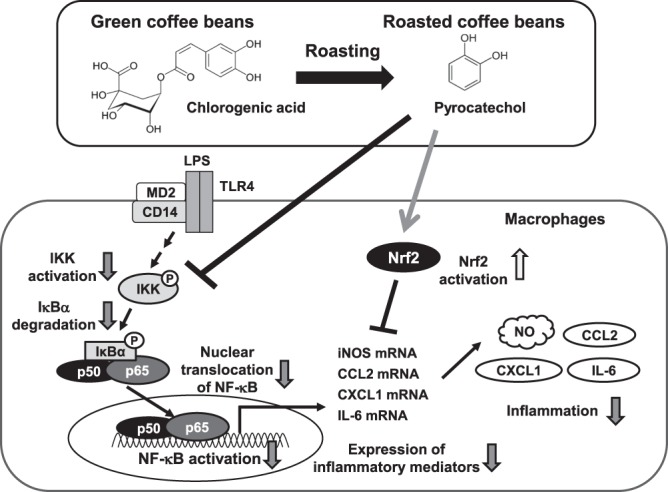
Pyrocatechol, a component of roasted coffee, exhibits anti-inflammatory activity. Pyrocatechol is formed from chlorogenic acid in coffee beans by roasting. Pyrocatechol inhibits LPS-induced NF-κB activation and induces Nrf2 activation, which negatively regulates LPS-induced inflammatory responses. As a result, pyrocatechol in roasted coffee beans suppresses NO production by inhibiting the mRNA expression of iNOS as well as CCL2, CXCL1, and IL-6.

In the present study, anti-inflammatory activity was observed in an extract of roasted coffee beans. Previous studies reported that coffee extracts contain many bioactive compounds, such as caffeine, diterpenes and several polyphenols^22,23^. To identify the ingredients in coffee extract, we evaluated the anti-inflammatory activities of several compounds that are regarded as medicinal ingredients. We examined the anti-inflammatory activities of caffeine, caffeic acid, trigonelline, and chlorogenic acid, and the results obtained showed that they did not suppress NO production or the secretion of CCL2 (Supplemental Fig. 7A,C). We also found that these coffee-derived compounds did not alter the LPS-induced mRNA expression of iNOS or CCL2 (Supplemental Fig. 7B,D). We demonstrated that pyrocatechol in roasted coffee beans, but not green beans exhibited anti-inflammatory activity (Fig. 15). A previous study reported that pyrocatechol was released from chlorogenic acid under reactive conditions (250 °C, 30 min)^31^, which is similar to the conditions for roasting coffee beans. This finding showed that a number of procedures, such as the roasting of coffee beans, results in the formation of medicinal ingredients. It is well known that the roasting process of coffee beans causes Maillard reaction, a chemical reaction between amino acids and reducing saccharides. Maillard reaction produces numerous compounds such as melanoidins^32^. Previously, Daglia *et al*. showed that melanoidin fractions in coffee exerted antioxidant activity and protected lipid peroxidation^33^. Therefore, it is possible that melanoidins are also involved in anti-inflammatory activity of coffee.

We showed that pyrocatechol significantly inhibited the expression of inflammatory cytokines such as IL-6 and TNFα but did not affect the expression of IL-10 (Fig. 10, Supplemental Fig. 5). Therefore, these results suggest that pyrocatechol exhibits anti-inflammatory effect through inhibiting the expression of inflammatory cytokines but not enhancing the expression of anti-inflammatory cytokines. We also investigated the anti-inflammatory effect of a nonsteroidal anti*-*inflammatory drug, indomethacin. As shown in Supplemental Fig. 8, high-concentrated indomethacin at 200 μM inhibited LPS-induced inflammatory responses such as NO production, mRNA expression of iNOS, CCL2, CXCL1, IL-6, and TNFα but not IL-10. Compared to the anti-inflammatory activity of indomethacin, it is suggested that pyrocatechol, a coffee component, is a potent anti-inflammatory agent exhibiting anti-inflammatory activity at lower concentration rather than the case of indomethacin. However, the direct target protein in the LPS signaling pathway for pyrocatechol has not yet been identified. As shown in Fig. 3B, the IKK complex appears to be one of the target molecules for coffee extract-induced anti-inflammatory effects. Furthermore, we investigated whether coffee extract affected the activation of NF-κB induced by another stimulation, such as TNFα; however, coffee extract did not affect the TNFα-induced activation of NF-κB in NIH-3T3 cells stably expressing the NF-κB-dependent luciferase reporter plasmid (Supplemental Fig. 9).

These results indicate that the target molecule for coffee extract, possibly for pyrocatechol, is present in the IKK complex activated by LPS, but not TNFα. The LPS-induced IKK complex includes several signaling proteins, such as transforming growth factor β-activated kinase 1 (TAK1), as a platform for complex formation, including MyD88, IL-1 receptor-associated kinase (IRAK) 1, IRAK4, TRAF6, and IKKγ, but does not depend on the kinase activity of TAK1^34^. However, the kinase activity of TAK1 is required for the LPS-induced activation of p38 and JNK^35^, suggesting that coffee extract does not affect the activity of TAK1 induced by LPS.

Furthermore, the coffee extract and pyrocatechol-mediated inhibition of LPS-induced inflammatory responses depended on the activation of another transcription factor, Nrf2 (Figs. 5 and 11). Previous studies reported functional crosstalk between NF-κB and Nrf2 in the LPS signaling pathway. Studies on Nrf2-deficient murine embryonic fibroblasts (Nrf2^−/−^MEF) revealed enhanced LPS-induced IKK activity that augmented the phosphorylation of IκBα and its subsequent degradation, indicating that Nrf2 inhibits the NF-κB activation pathway^36^. We also showed that expression of Nrf2 negatively regulated LPS-induced IKK activation and nuclear localization of NF-κB (Fig. 5C,D). Nrf2 may suppress the IKK activation through the expression of target genes of Nrf2. However, the target gene of Nrf2 that is critical for suppressing the activation of IKK has not yet been identified.

The mechanisms by which coffee extract and pyrocatechol induce the activation of Nrf2 remain unclear. Under basal unstressed conditions, Nrf2 is constitutively ubiquitinated by Kelch-like ECH-associated protein 1 (Keap1), an adaptor component of the cullin 3 (Cul3)-based ubiquitin E3 ligase complex, resulting in its proteasomal degradation^37^. A previous study reported that an increased level of ROS promotes the dissociation of Nrf2 and Keap1, either via the activation of kinases that phosphorylate Nrf2 or by the oxidization of key cysteine residues that govern Keap1 activity^38^. The present results showed that the coffee extract treatment increased intracellular ROS levels in RAW264.7 cells (data not shown); therefore, coffee extract may inhibit the ubiquitin E3 ligase activity of the Keap1-Cul3 complex by increasing ROS levels, leading to the stabilization of Nrf2. When RAW264.7 cells were treated with coffee extract, Nrf2 activated its target genes including the detoxification and antioxidant enzymes heme oxygenase-1 (HO-1) and NAD(P)H quinone dehydrogenase 1 (NQO1) (Supplemental Fig. 3). Kil *et al*. reported that the knockdown of HO-1 using siRNA reduced the inhibitory effects of okanin, a flavonoid, on LPS-induced NO production and iNOS expression in RAW264.7 cells^39^. Additionally, silencing the expression of NQO1 alone, or in combination with HO-1, markedly increased LPS-induced TNFα and IL-1β expression^40^. These findings indicate that HO-1 and NQO1 induced by coffee extract via Nrf2 expression modulate LPS-induced inflammatory responsiveness. Additionally, we noted that coffee extract inhibited IKK by positively decelerating its kinase activity from control conditions (Fig. 3B). This suggests the presence of negative regulators, such as protein phosphatase against the IKK complex, and that coffee extract enhances the activities of negative regulator for IKK.

The identification of pyrocatechol as a medicinal ingredient in coffee extract is very important, and the further identification of target proteins for pyrocatechol will be crucial for clarifying the mechanisms underlying coffee-induced anti-inflammatory effects. As shown in Fig. 9, R3 fraction also exhibited the inhibitory effect against LPS stimulation. Until now, we found that 4-ethylcatechol is contained in R3 fraction. Although the quantity of 4-ethylcatechol and its anti-inflammatory activity were not investigated, 4-ethylcatechol could be an active ingredient in R3 (Matsutaka *et al*. Unpublished data). To understand the total picture of the anti-inflammatory activity of coffee, it will be necessary to analyze a generating mechanism of 4-ethylcatechol during roasting process of coffee and clarify anti-inflammatory mechanism of 4-ethylcatechol in the future.

## Methods

### Preparation of coffee extract

Coffee (Columbia Arabica) roasted at 220 °C for 20 min was obtained from Starbucks Coffee Japan (Tokyo, Japan). Green coffee beans (Columbia Arabica) were obtained from Yumekobo (Tokyo, Japan) and roasted at 220 °C for the indicated periods using Coffee Roaster MR-101 (DAINICHI, Niigata, Japan). Preparation of extracts of green coffee beans and roasted coffee beans were performed as described below; 8 g coffee powder was poured with 140 mL of hot water (95 °C), and the coffee grounds were removed by passing through filter paper (Mellita, Minden, Germany). Then, the coffee extracts were aliquoted into small volumes, and stored at −80 °C until used. According to previous report^38^, the concentration of dry weight in undiluted 100% (v/v) coffee extract was determined as 8.4 mg/mL.

### Reagents and antibodies

LPS (*Escherichia coli* 055: B5) and an anti-GST antibody were purchased from Sigma-Aldrich (St. Louis, MO, USA) and Nacalai Tesque (Kyoto, Japan), respectively. Anti-NF-κB, anti-Nrf2, anti-p38, anti-iNOS, anti-IKKγ, anti-Lamin B, and anti-β-actin antibodies were supplied by purchased from Santa Cruz Biotechnology (Santa Cruz, CA, USA). All other antibodies were from Cell Signaling Technology (Danvers, MA, USA). Other reagents were purchased from Nacalai Tesque.

### Cell culture and the treatment with LPS

The murine macrophage cell line, RAW264.7 was purchased from the Riken Cell Bank (Ibaraki, Japan) and cultured in DMEM (Nacalai Tesque) with 10% fetal bovine serum (FBS) (Gibco, Life Technologies, CA, USA) and 1% Penicillin-Streptomycin Mixed Solution (Nacalai Tesque). As a result having performed the LPS does and time dependent experiment as previously reported^41^, RAW264.7 cells were treated with LPS (1 μg/mL) for suitable periods to evaluate each reaction.

### Measurement of cell viability

RAW264.7 cells (5 × 10^5^ cells) were seeded in a 24-well plate and preincubated with coffee extract or pyrocatechol at 37 °C for 1 h prior to the stimulation with LPS (1 μg/mL) for 24 h. The trypan blue dye exclusion test was used to determine cell viability.

### Measurement of NO

RAW264.7 cells (2 × 10^5^ cells) were seeded in a 24-well plate and preincubated with coffee extract or pyrocatechol at 37 °C for 1 h prior to the stimulation with LPS (1 μg/mL) for 24 h. The culture medium was collected and nitrite was measured by the Griess reaction as previously described^41^.

### Immunoblotting

After the desired time of incubation, cells were lysed in NP-40 lysis buffer (50 mM Tris-HCl (pH 8.0), 120 mM NaCl, 1 mM EDTA (pH8.0), 0.5% Nonidet P-40, 10 mM β-glycerophosphate, 2.5 mM NaF, 0.1 mM Na_3_VO_4_, 2 μg/mL aprotinin, and 2 μg/mL leupeptin). Cell lysates were incubated on ice for 10 min, centrifuged, and the resulting supernatant was mixed with Laemmli’s sample buffer. Preparation of nuclear extracts were performed as described previsouly^42^. Denatured samples were resolved by SDS-PAGE and transferred to polyvinylidene difluoride membranes (Millipore, Billerica, MA, USA). The bound antibodies on PVDF membrane were detected using peroxidase-conjugated secondary antibodies (Dako-Japan, Tokyo, Japan), and visualized by the ECL detection system (GE Healthcare, Little Chalfont, UK). The intensity of each band was quantified by ImageJ software.

### Enzyme-linked immunosorbent assay (ELISA)

RAW264.7 cells (2 × 10^5^ cells) seeded in a 24-well plate were preincubated with coffee extract or pyrocatechol at 37 °C for 1 h prior to the stimulation with LPS (1 μg/mL) for 24 h. Murine serum was collected at 1 hour after LPS injection as described below. The amounts of CCL2 and IL-6 in the supernatants of RAW264.7 cells and in the murine serum were determined using the Immunoassay Kits (eBioscience, San Diego, CA, USA) and CXCL1 ELISA kit (R&D Systems, Minneapolis, MN, USA).

### Transfection of siRNA

RAW264.7 cells (5 × 10^6^) were transfected with 10 μM control siRNA or siRNA targeting murine Nrf2 (GE Healthcare UK Ltd, United Kingdom) using DharmaFECT (GE Healthcare UK Ltd). Cell lysates and RNA were prepared 48 h post-transfection.

### Luciferase assay

RAW264.7 cells (2 × 10^5^ cells) were transfected with 0.2 μg of pNF-κB-Luc (Promega, Madison, WI, USA) and 0.2 μg of pRL-TK (Promega) using the Neon® Transfection System (Life Technologies). After 36 h, transfected cells were treated with coffee extract or pyrocatechol for 1 h prior to the stimulation with LPS (1 μg/mL) for 6 h. Luciferase assay was performed using the Dual-Luciferase Reporter Assay System (Promega)^42^.

### *In vitro* IKK assay

Cell lysates were prepared using NP-40 lysis were immunoprecipittated with 1 μg of an anti-IKKγ antibody and 20 μL protein G agarose (Zymed Laboratory, South San Francisco, CA) at 4 °C for 2 h. After immunoprecipitation, kinase assay was performed using 1 μg of GST-IκBα were performed as previously described^43^. To evaluate the relative IKK activity, the phosphorylation level of GST-IκBα was normalized with the amounts of GST-IκBα to show the relative IKK activity.

### RT-PCR (reverse transcription-polymerase chain reaction)

RNA extraction, Reverse-transcriptase (RT) reaction and Quantitative real-time PCR were performed as described previously^42^. The PCR primer sequences used were as follows:

iNOS:5′-TCTGCGCCTTTGCTCATGAC-3′ (upstream) and 5′-TAAAGGCTCCGGGCTCTG-3′ (downstream), CCL2: 5′-TGAGGTGGTTGTGGAAAAGG-3′ (upstream) and 5′-CCTGCTGTTCACAGTTGCC-3′ (downstream), CXCL1: 5′-TGGGGACACCTTTTAGCATC-3′ (upstream) and 5′-GCCCATCGCCAATGAGCTG-3′ (downstream), IL-6: 5′-CCAGAGATACAAAGAAATGATGG-3′ (upstream) and 5′-ACTCCAGAAGACCAGAGGAAAT-3′ (downstream), IL-10: 5′-ATTTGAATTCCCTGGGTGAGAAG-3′ (upstream) and 5′-CACAGGGGAGAAATCGATGACA-3′ (downstream), GAPDH: 5′-ACTCCACTCACGGCAAATTC-3′ (upstream) and 5′-CCTTCCACAATGCCAAAGTT-3′ (downstream).

### LPS-induced inflammation in mice

Male C57BL/6JJmsSlc mice (4 weeks old) were obtained from Sankyo Labo Service Corporation, Inc. (Tokyo, Japan). For each experiment to measure secretion level of cytokines in serum and measure the mRNA expression level of cytokines, twenty-four mice were divided into two groups and given pure water or 60% (v/v) coffee extract for 3 weeks. Twenty-four mice were divided into two groups and given pure water or 74.4 μM pyrocatechol for 3 weeks. Mice were allowed free access to food, water, coffee extract, and pyrocatechol solution. Body weights and the amounts of food consumed and water, coffee extract, and pyrocatechol solution drank were measured twice a week for 3 weeks. Mice in each group were further randomly divided into two groups containing 6 mice each. Mice were injected intraperitoneally with control vehicle (PBS) or LPS (20 mg/kg). Then, 1 h after the injection of LPS, blood samples were collected from mice by cardiac puncture and serum samples were separated. Mice were sacrificed 7 h after the injection of LPS, and total RNA was extracted from the kidneys, liver, and lungs using Trizol (life technologies). Each *in vivo* experiment was performed on time using 6 mice in each group. All experimental protocols were approved by the Animal Usage Committee of Keio University (Approval number, 12048-(2)). Methods were performed in accordance with the approved guidelines.

### Extraction and separation of coffee extract and high-performance liquid chromatography (HPLC)

To extract coffee constituents with organic solvents, brewed coffee (1 mL) was extracted sequentially with equal volumes of hexane, ethyl acetate, and 1-butanol. Organic and aqueous phases were then concentrated by evaporation, and the concentrates were dissolved in 20 µL DMSO. To separate ethyl acetate extracts, dried ethyl acetate extracts (equivalent to 15 mL coffee) were dissolved in 15 mL of methanol/ethyl acetate (2:1), applied to a Sep-Pak C18 column (Waters, Milford, MA), and eluted by 15-mL stepwise rinses of water (R1), 20% methanol (R2), and 100% methanol (R3). High‐performance liquid chromatography (HPLC) analyses were performed using an ODS column (Inertsil ODS‐3 column, 4.6 × 250 mm, GL Sciences Inc., Tokyo, Japan) at 40 °C. Coffee extracts or standards were applied to the column, eluted with the solvent A (5% acetate) to B (100% methanol) gradient system at a flow rate of 1 mL min^−1^, and detected with fluorescence at λex = 280 nm and λem = 320 nm.

### Extraction and separation of coffee extract and high-performance liquid chromatography (HPLC)

The brewed coffee was extracted sequentially with organic solvents and the ethyl acetate extracts were further fractioned using the ODS column into three fractions: R1, R2, and R3, as described previously^43^. High‐performance liquid chromatography (HPLC) analyses were performed using an ODS column (Inertsil ODS‐3 column, 4.6 × 250 mm, GL Sciences Inc., Tokyo, Japan) at 40 °C as described previously^44^.

### Statistical analysis

The results are expressed as means ± SD from three independent experiments. A one- or two-way analysis of variance (ANOVA) followed by Tukey’s test was used to evaluate differences between more than three groups. Differences were considered significant when *p* < 0.05.

## Supplementary information

Supplementary figures.
