# Supplementary material for: Pyrocatechol, a component of coffee, suppresses LPS-induced inflammatory responses by inhibiting NF-κB and activating Nrf2
Source: Sci Rep. 2020 Feb 13;10:2584. doi: 10.1038/s41598-020-59380-x (PMC7018815; doi:10.1038/s41598-020-59380-x)

Supplement information

Pyrocatechol, a component of coffee, suppresses LPS-induced inflammatory responses by inhibiting NF-κB and activating Nrf2

Megumi Funakoshi-Tago[a], Yusuke Nonaka[a], Kenji Tago[b] Mika Takeda[a], Yuma Ishihara[a], Ami Sakai, Mari Matsutaka, Kenji Kobata, and Hiroomi Tamura[a]

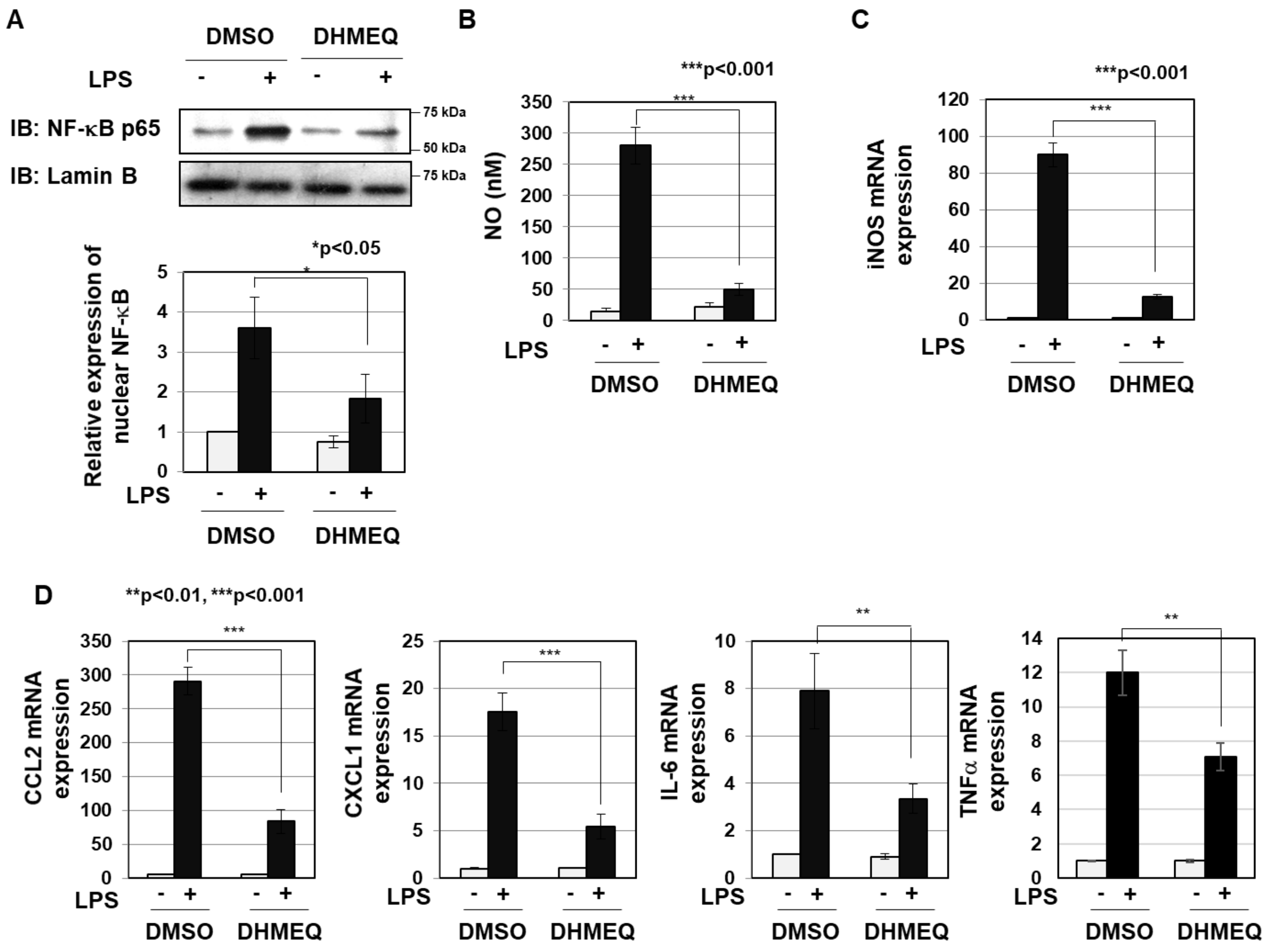

Supplemental Figure 1 The NF-κB inhibitor, DMMEQ inhibited LPS-induced inflammatory responses.

RAW264.7 cells (5×10^6 cells) were pretreated with DMSO (0.1%) and DHMEQ (10 μM) for 1 h prior to the stimulation with LPS (1 μg/mL). (A) Nuclear extracts were immunoblotted with an anti-NF-κB p65 or anti-Lamin B antibody 30 min after the LPS stimulation. The relative expression levels of NF-κB in the nucleus are shown in the graphs. *$p<0.05$ significantly different from control cells stimulated with LPS. (B) Nitrate concentrations in culture supernatants were measured 24 h after the LPS stimulation using Griess reagent. ***$p<0.001$ significantly different from control cells treated with LPS. (C) iNOS mRNA expression was assessed 12 h after the LPS stimulation by RT-PCR. GAPDH mRNA expression was used as an internal control.

***$p<0.001$ significantly different from control cells treated with LPS. (D) The mRNA expression of CCL2, CXCL1, IL-6, and TNFα was assessed 2 h after the LPS stimulation by RT-PCR. GAPDH mRNA expression was used as an internal control. **$p<0.01$, ***$p<0.001$ significantly different from control cells treated with LPS.

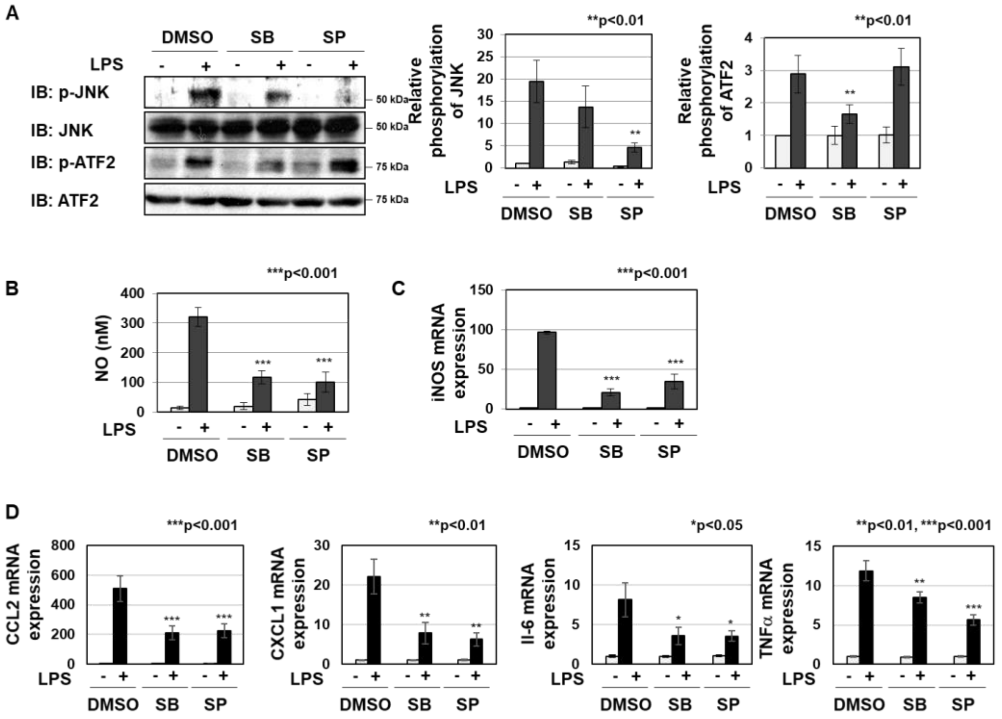

Supplemental Figure 2 The p38 inhibitor, SB203580 and JNK inhibitor, SP600125 both inhibited LPS-induced inflammatory responses.

RAW264.7 cells (5×10^6 cells) were pretreated with DMSO (0.1%), SB203580 (SB) (10 μM), and SP600125 (SP) (10 μM) for 1 h prior to the stimulation with LPS (1 μg/mL). (A) Whole cell lysates were immunoblotted 30 min after the LPS stimulation with an anti-phospho-JNK, anti-JNK, anti-phospho-ATF2, or anti-ATF2 antibody. The relative phosphorylation of JNK and ATF2 is shown in the graphs. Values are given as means ± SD from three independent experiments. **$p<0.01$ significantly different from control cells stimulated with LPS. (B) Nitrate concentrations in culture supernatants were measured 24 h after the LPS stimulation using Griess reagent. ***$p<0.001$ significantly different from control cells treated with LPS. (C) iNOS mRNA expression was assessed 12 h after the LPS stimulation by RT-PCR. GAPDH mRNA expression was used as an internal control. ***$p<0.001$ significantly different from control cells treated with LPS. (D) The mRNA expression of CCL2, CXCL1, IL-6, and TNFα was assessed 2 h after the LPS stimulation by RT-PCR. GAPDH mRNA expression was used as an internal

control. *p<0.05, **p<0.01, ***p<0.001 significantly different from control cells treated with LPS.

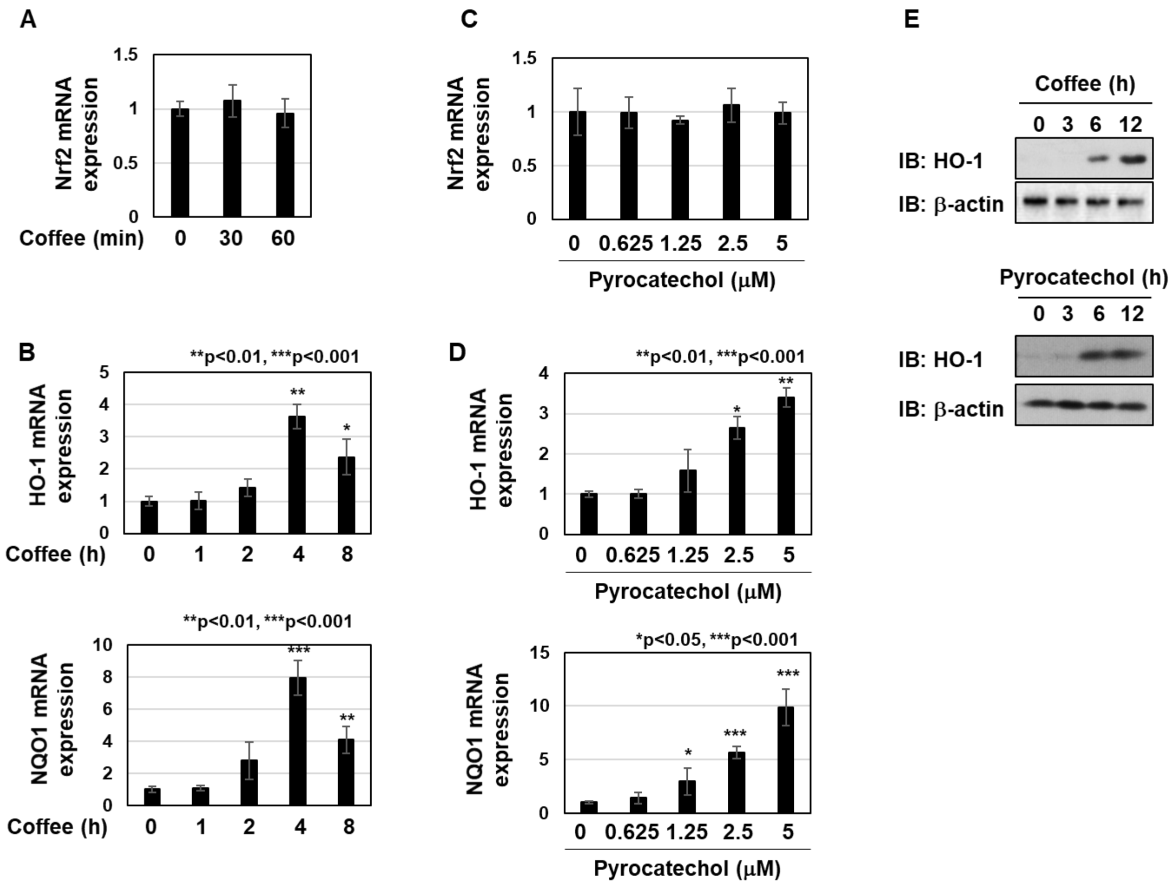

Supplemental Figure 3 Coffee extract and pyrocatechol induces the mRNA expression of HO-1 and NQO1, but not Nrf2.

(A, B) RAW264.7 cells (5×10^6 cells) were treated with coffee extract (5% (v/v)) for the indicated periods. The mRNA expression of Nrf2, HO-1, and NQO1 was assessed by RT-PCR. GAPDH mRNA expression was used as an internal control. The PCR primer sequences used were as follows: Nrf2: 5′-CCAGAAGCCACACTGACAGA-3′ (upstream) and 5′-GGAGAGGATGCTGCTGAAAG-3′ (downstream), HO-1: 5′-CACGCATATACCCGCTACCT-3′ (upstream) and 5′-CCAGAGTGTTCATTCGAGCA-3′ (downstream), NQO1: 5′-TTCTCTGGCCGATTCAGAGT-3′ (upstream) and 5′-GGCTGCTTGGAGCAAAATAG-3′ (downstream), GAPDH: 5′-ACTCCACTCACGGCAAATTC-3′ (upstream) and 5′-

CCTTCCACAATGCCAAAGTT-3′ (downstream). *$p<0.05$, **$p<0.01$,***$p<0.001$ significantly different from control cells. (C, D) RAW264.7 cells (5×10^6 cells) were treated with pyrocatechol (0.625, 1.25, 2.5, and 5 μM) for 4 h. The mRNA expression of Nrf2, HO-1, and NQO1 was assessed by RT-PCR. GAPDH mRNA expression was used as an internal control. *$p<0.05$, **$p<0.01$,***$p<0.001$ significantly different from control cells.(E) RAW264.7 cells (5×10^6 cells) were treated with coffee extract (5% (v/v)) or pyrocatechol (2.5 μM) for the indicated periods. Whole cell lysates were immunoblotted with anti-HO-1 antibody or anti-β-actin antibody.

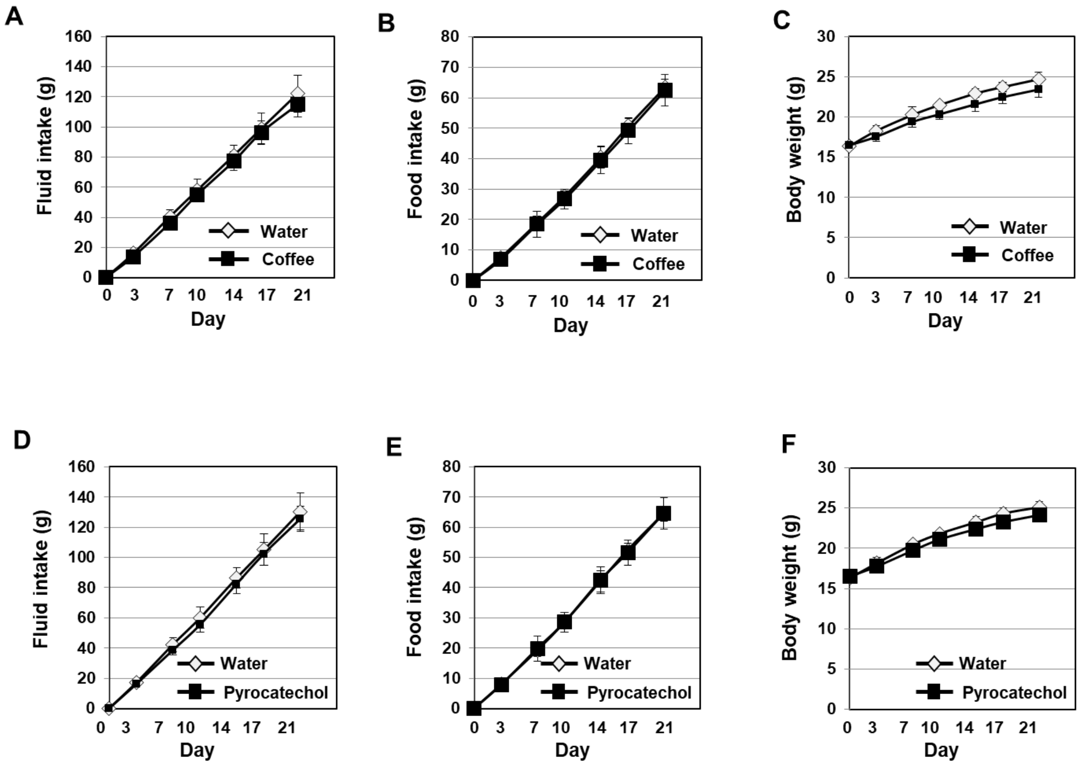

Supplemental Figure 4 Intake of coffee and pyrocatechol did not affect food consumption amounts or increases in body weight.

(A-C) Male C57BL/6 mice were given water or 60% (v/v) coffee extract for 3 weeks (n = 12 mice/group) (D-F) Male C57BL/6 mice were given 77.4 μM pyrocatechol for 3 weeks (n = 12 mice/group). Cumulative drinking intake (A, D), food consumption (B, E) and body weights (C, F) were graphed.

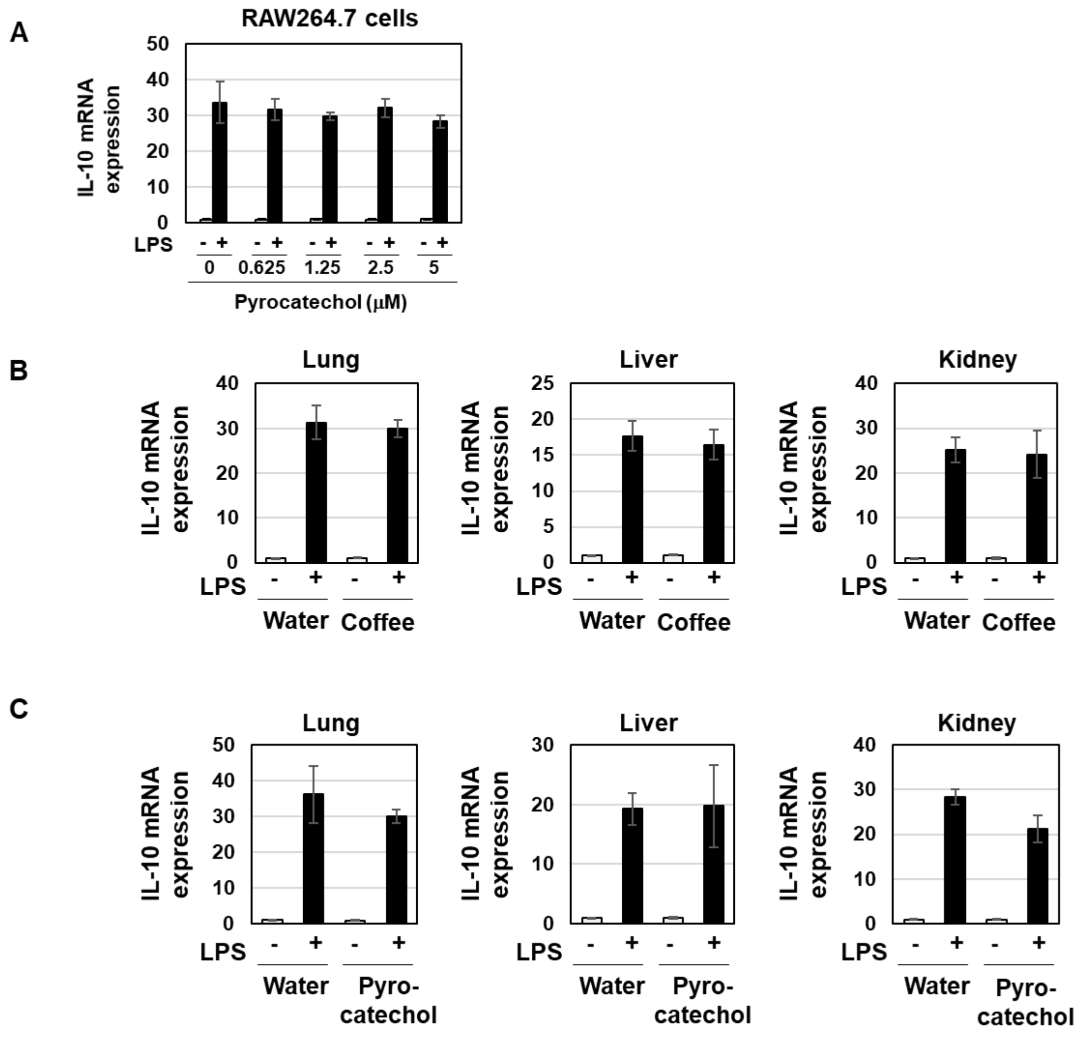

Supplemental Figure 5 Pyrocatechol had no effect on LPS-induced expression of IL-10 mRNA in RAW264.7 cells and mice injected with LPS.

(A) RAW264.7 cells (5×10^6 cells) were pretreated with pyrocatechol (0.625, 1.25, 2.5, and 5 μM) for 1h prior to the stimulation with LPS (1 μg/mL) for 2 h. (B, C) Male C57BL/6 mice were given water, 60% (v/v) coffee or 74.4 μM pyrocatechol for 3 weeks and then injected intraperitoneally with PBS or LPS (200 μg/mouse) (6 mice per group). Seven hours after the injection of PBS or LPS, total RNA was extracted from the lungs, liver, and kidneys. (A-C) The mRNA expression of IL-10 mRNA was assessed by RT-PCR. GAPDH mRNA expression was used as an internal control.

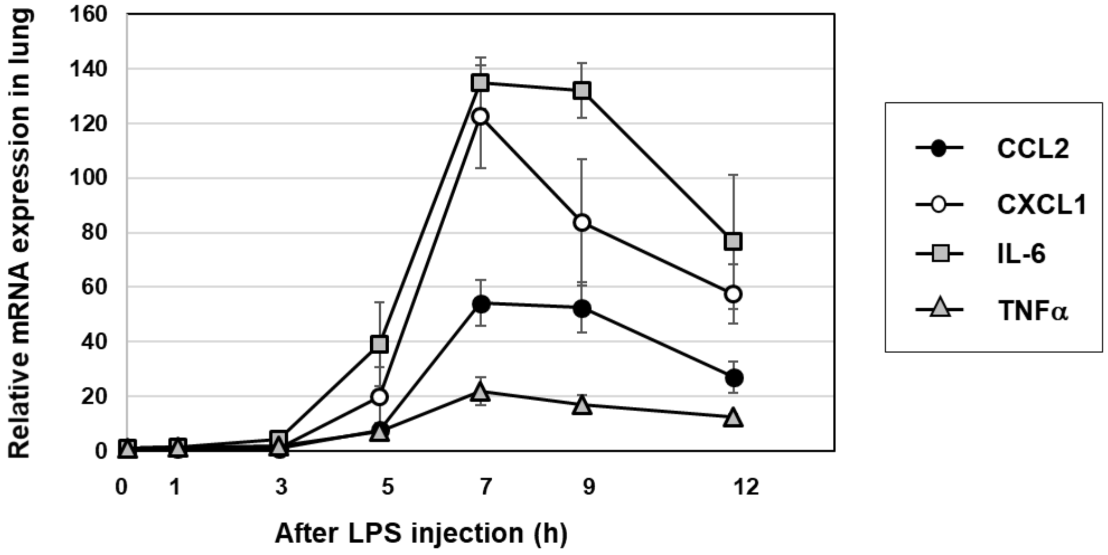

Supplemental Figure 6 The mRNA expression of CCL2, CXCL1, IL-6 and TNFα was induced in lung of C58BL/6 mice injected with LPS.

Male C57BL/6 mice were injected intraperitoneally with LPS (200 μg/mouse) (n=4). Total RNA was extracted from the lungs and the mRNA expression of CCL2, CXCL1, IL-6 and TNFα was assessed by RT-PCR. GAPDH mRNA expression was used as an internal control.

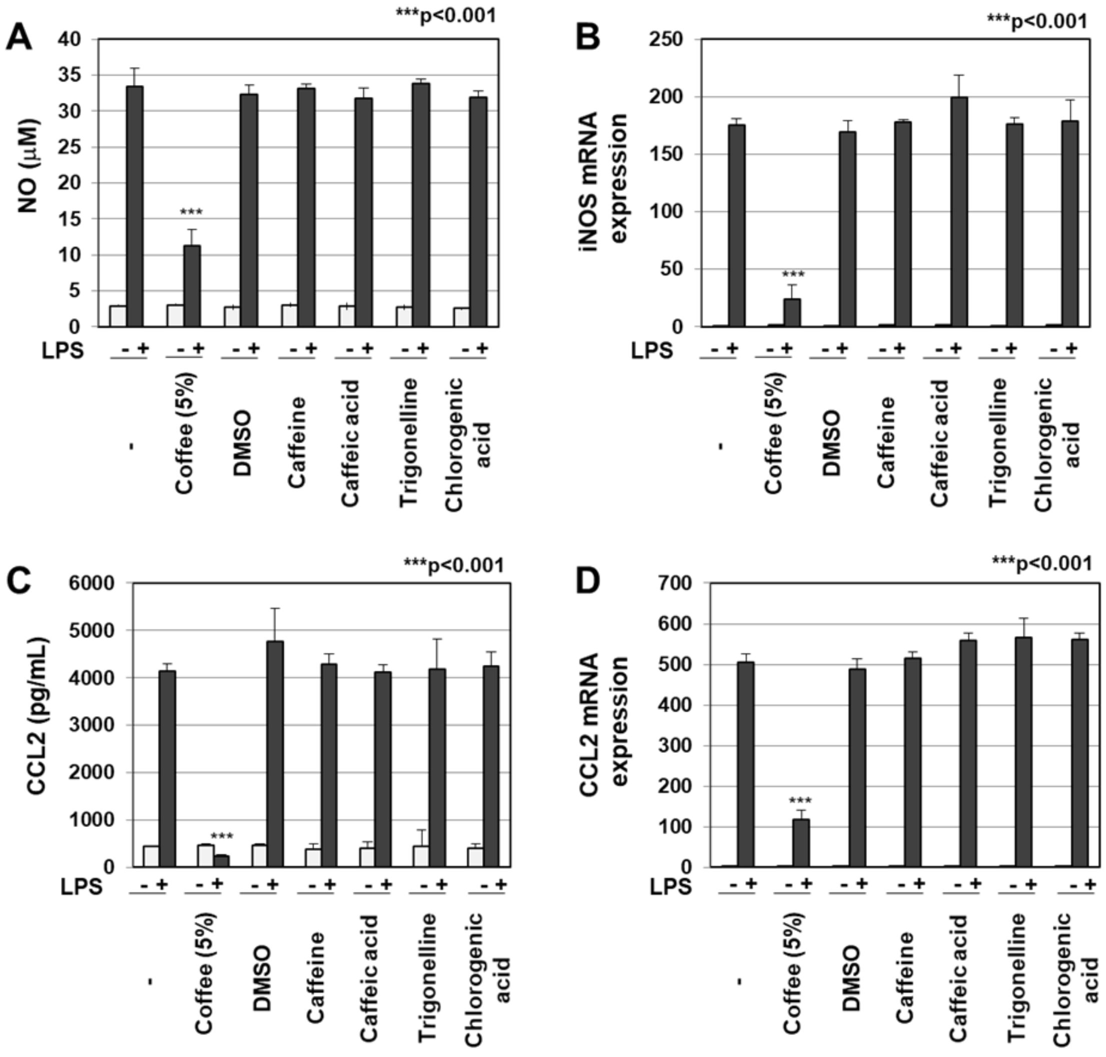

Supplemental Figure 7 Caffeine, caffeic acid, trigonelline, and chlorogenic acid had no effect on LPS-induced inflammatory responses.

(A) RAW264.7 cells (5×10^6 cells) were pretreated with roasted coffee extract (5%(v/v)), DMSO (0.1%), caffeine (100 μM), caffeic acid (100 μM), trigonelline (100 μM), and chlorogenic acid (100 μM) for 1 h prior to the stimulation with LPS (1 μg/mL). (A) Nitrate concentrations in culture supernatants were measured 24 h after the LPS stimulation using Griess reagent. ***p<0.001 significantly different from control cells treated with LPS. (B) iNOS mRNA expression was assessed 12 h after the LPS stimulation by RT-PCR. GAPDH mRNA expression was used as an internal control.

***$p<0.001$ significantly different from control cells treated with LPS. (C) The amounts of CCL2 in supernatants were evaluated 24 h after the LPS stimulation by ELISA. ***$p<0.001$ significantly different from control cells treated with LPS. (D) CCL2 mRNA expression was assessed 2 h after the LPS stimulation by RT-PCR. GAPDH mRNA expression was used as an internal control. ***$p<0.001$ significantly different from control cells treated with LPS.

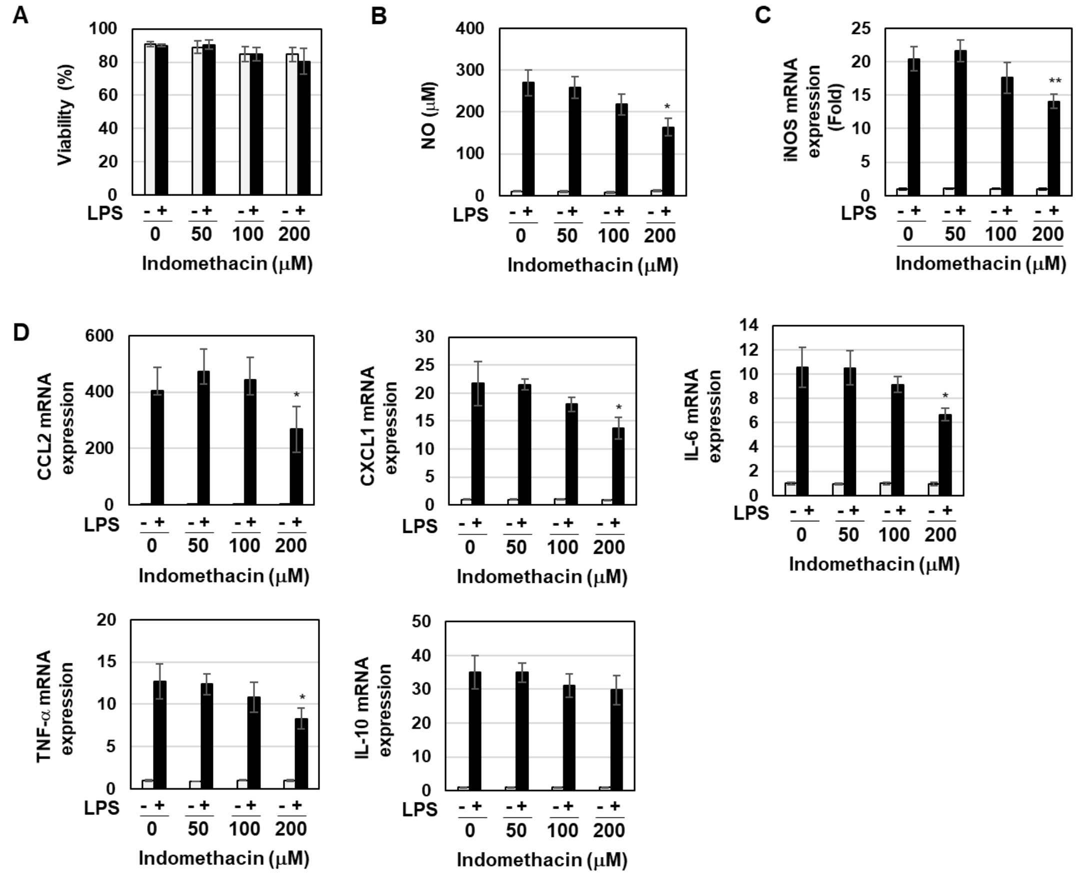

Supplemental Figure 8 Indomethacin at 200 μM inhibited the LPS-induced inflammatory responses.

RAW264.7 cells (5×10^{6} cells) were pretreated with indomethacin (50, 100, 200 μM) for 1 h prior to the stimulation with LPS (1 μg/mL). (A) The cell viability was analyzed 24 h after the LPS stimulation using trypan blue exclusion tests. (B) Nitrate concentrations in culture supernatants were measured 24 h after the LPS stimulation using Griess reagent. *p<0.05 significantly different from control cells treated with LPS. (C) iNOS mRNA expression was assessed 12 h after the LPS stimulation by RT-PCR. GAPDH mRNA expression was used as an internal control. **p<0.01 significantly different from control cells treated with LPS. (D) The mRNA expression of CCL2, CXCL1, IL-6, TNFα and IL-10 was 2 h after the LPS stimulation by RT-PCR. GAPDH mRNA expression was used as an internal control. *p<0.05 significantly different from control cells treated with LPS.

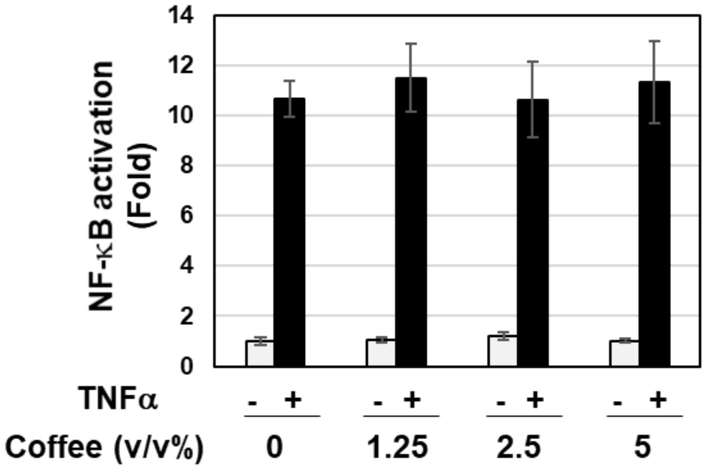

Supplemental Figure 9 Coffee had no effect on TNFα-induced NF-κB activation. NIH-3T3 cells stably expressing the NF-κB-dependent luciferase reporter plasmid (5×10^4 cells) were cultured in a 24-well plate and pretreated with the roasted coffee extract (1.25, 2.5, and 5% (v/v)) for 1 h prior to the stimulation with TNFα (10 ng/mL) for 6 h. After washing with PBS, cells were harvested in passive lysis buffer (Promega, Madison, WI, USA) and the luciferase activity of lysates was assessed using the luciferase reporter assay system (Promega). NF-κB-dependent luciferase activity was normalized by the quantity of protein for each sample.

Photographs of the full-length blots utilized in Figures (1)

Fig. 1 D

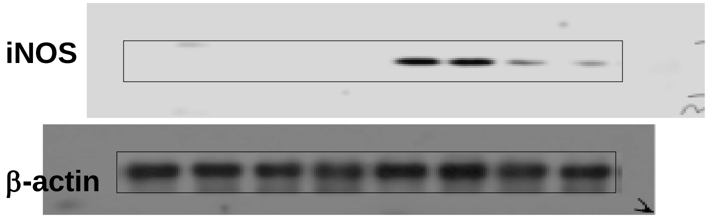

Fig. 3 B

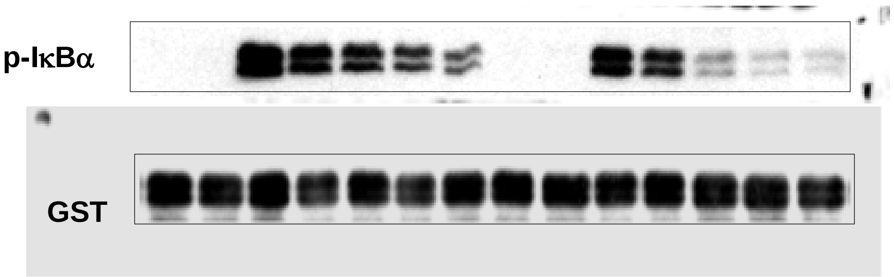

Fig. 3 C

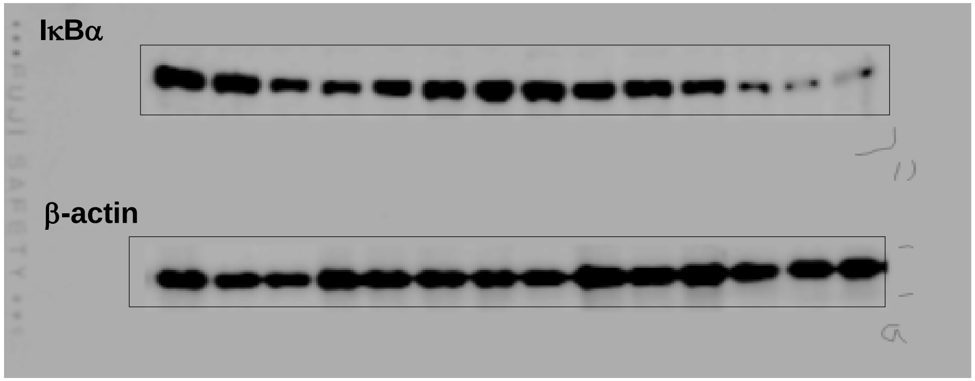

Fig. 3 D

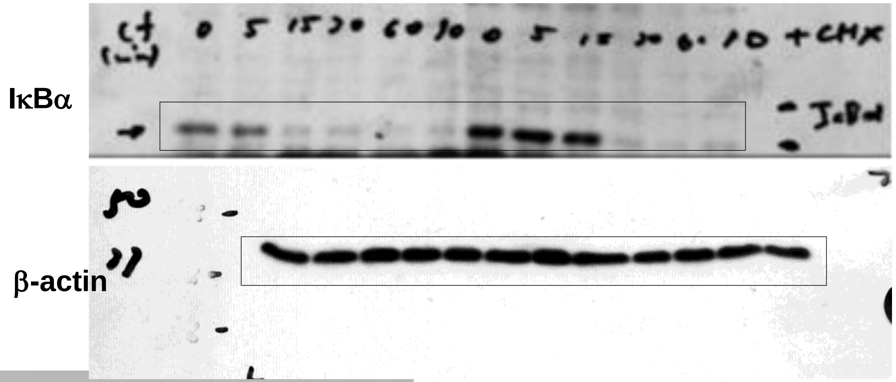

Fig. 3 E

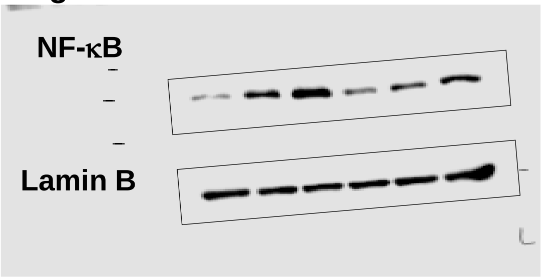

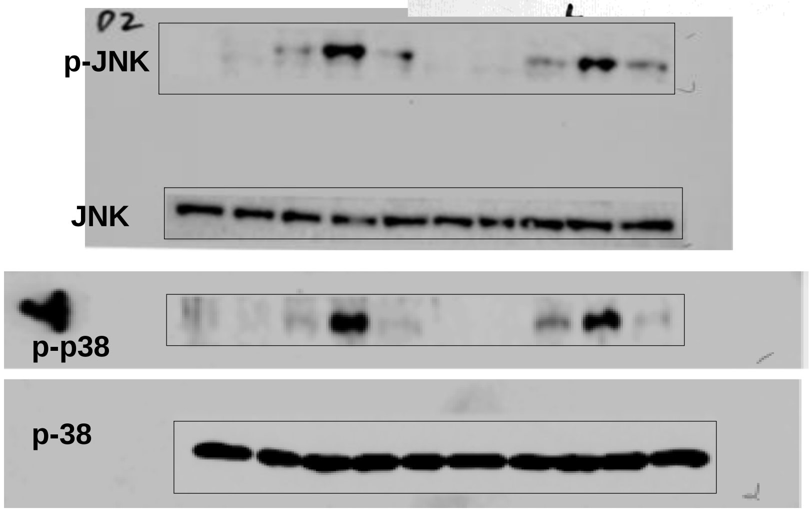

Photographs of the full-length blots utilized in Figures (2)

Fig. 5 A

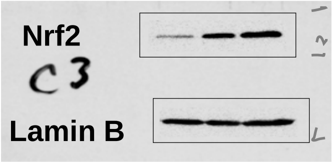

Fig. 5 B

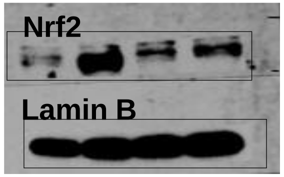

Fig. 5 C

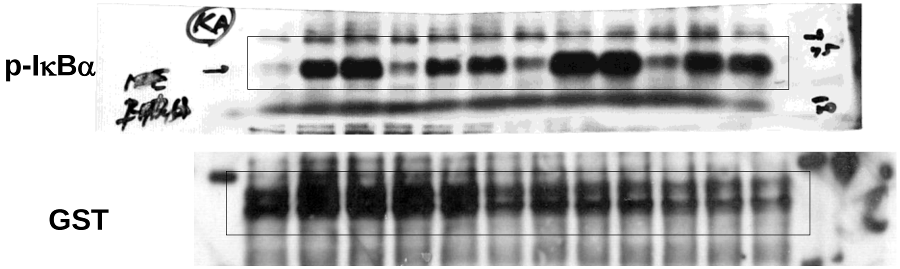

Fig. 5 D

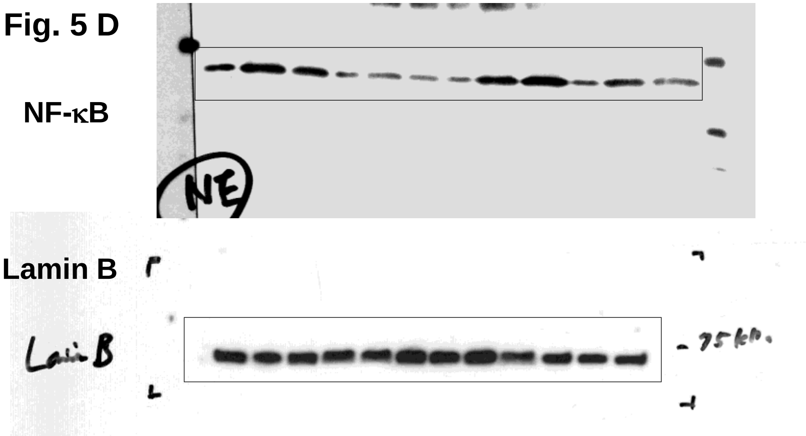

Fig. 7 G

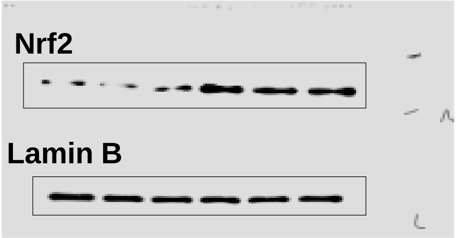

Fig. 8 H

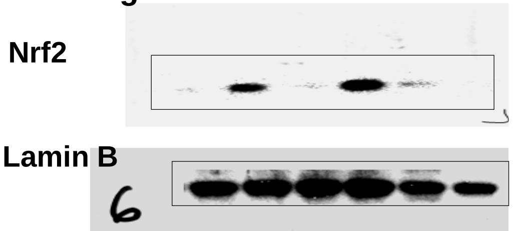

Fig. 9 H

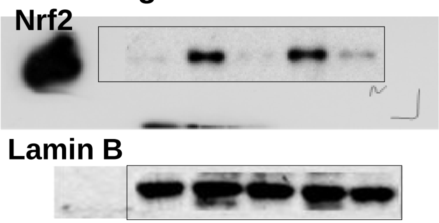

Fig. 11 B

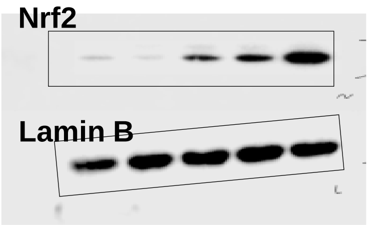

Photographs of the full-length blots utilized in Figures (3)

Supplemental Figure 1A

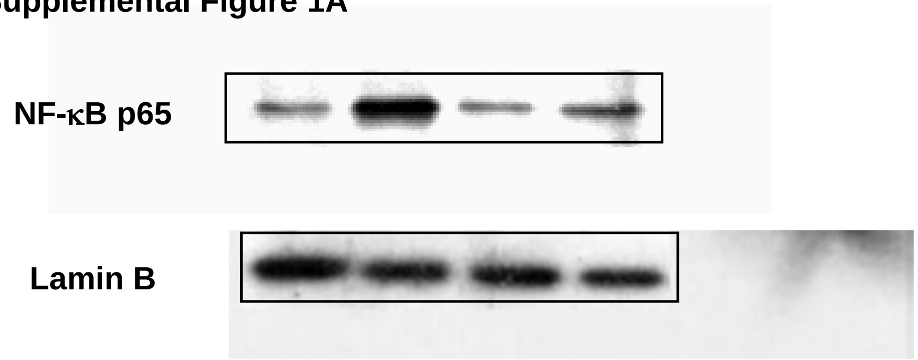

Supplemental Figure 3E

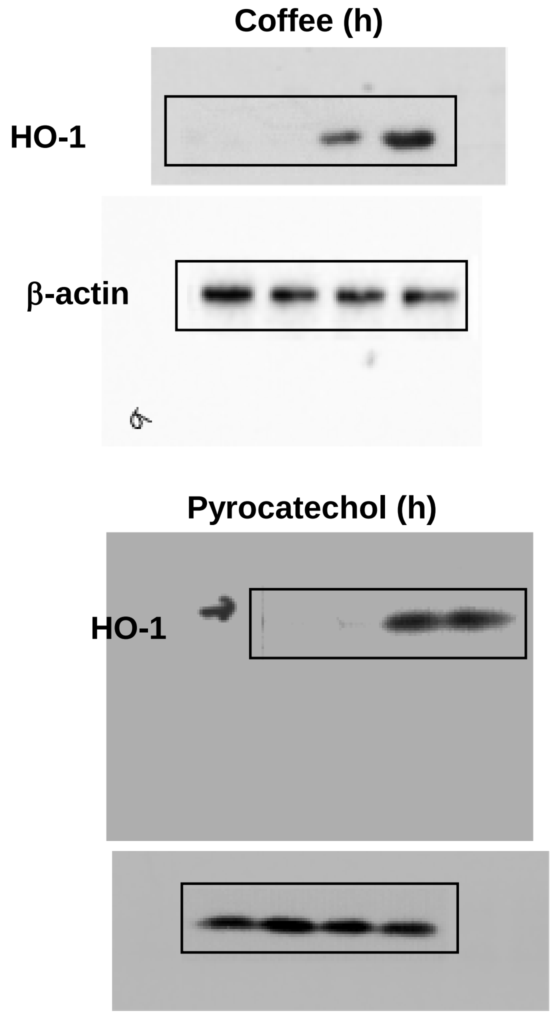

Supplemental Figure 2A

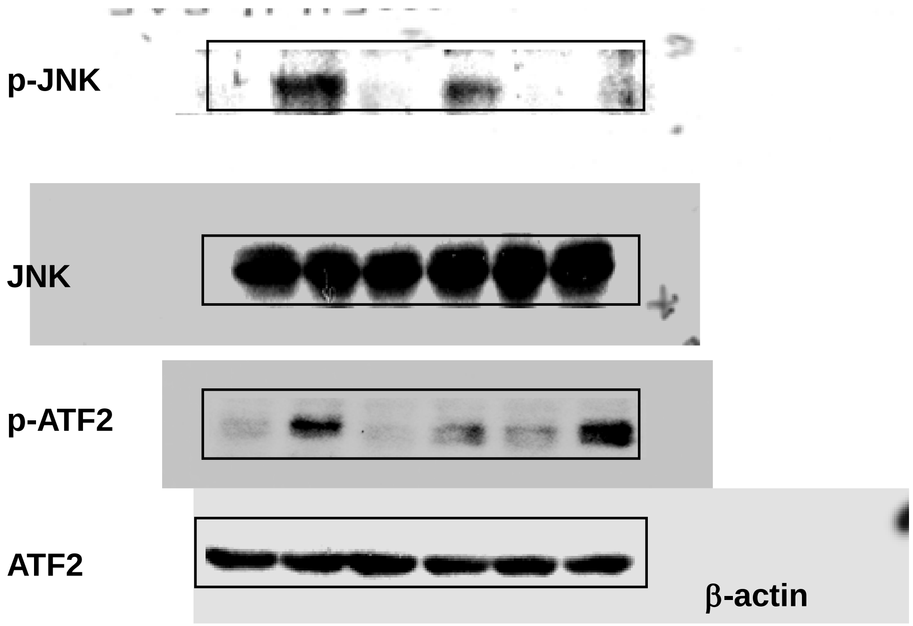

Supplement: Supplementary file 1 — Supplementary figures. [file 41598_2020_59380_MOESM1_ESM.pdf]
